# Assessing and mitigating privacy risks of sparse, noisy genotypes by local alignment to haplotype databases

**DOI:** 10.1101/gr.278322.123

**Published:** 2023-12

**Authors:** Prashant S. Emani, Maya N. Geradi, Gamze Gürsoy, Monica R. Grasty, Andrew Miranker, Mark B. Gerstein

**Affiliations:** 1 Program in Computational Biology and Bioinformatics, Yale University, New Haven, Connecticut 06520, USA;; 2 Department of Molecular Biophysics and Biochemistry, Yale University, New Haven, Connecticut 06520, USA;; 3 Department of Computer Science, Yale University, New Haven, Connecticut 06520, USA;; 4 Department of Statistics and Data Science, Yale University, New Haven, Connecticut 06520, USA

## Abstract

Single nucleotide polymorphisms (SNPs) from omics data create a reidentification risk for individuals and their relatives. Although the ability of thousands of SNPs (especially rare ones) to identify individuals has been repeatedly shown, the availability of small sets of noisy genotypes, from environmental DNA samples or functional genomics data, motivated us to quantify their informativeness. We present a computational tool suite, termed Privacy Leakage by Inference across Genotypic HMM Trajectories (PLIGHT), using population-genetics-based hidden Markov models (HMMs) of recombination and mutation to find piecewise alignment of small, noisy SNP sets to reference haplotype databases. We explore cases in which query individuals are either known to be in the database, or not, and consider several genotype queries, including those from environmental sample swabs from known individuals and from simulated “mosaics” (two-individual composites). Using PLIGHT on a database with ∼5000 haplotypes, we find for common, noise-free SNPs that only ten are sufficient to identify individuals, ∼20 can identify both components in two-individual mosaics, and 20–30 can identify first-order relatives. Using noisy environmental-sample-derived SNPs, PLIGHT identifies individuals in a database using ∼30 SNPs. Even when the individuals are not in the database, local genotype matches allow for some phenotypic information leakage based on coarse-grained SNP imputation. Finally, by quantifying privacy leakage from sparse SNP sets, PLIGHT helps determine the value of selectively sanitizing released SNPs without explicit assumptions about population membership or allele frequency. To make this practical, we provide a sanitization tool to remove the most identifying SNPs from genomic data.

Privacy concerns in the digital age are ubiquitous, with individual data collection, access, and the sophistication of tools of attack concurrently increasing to render individuals vulnerable to a significant risk of compromising data exposure. Incursions upon individual privacy include the removal of personal control over the uses of such data, and the possibility of being subjected to discrimination on the basis of revealed information. Perhaps the most invasive forms of such attacks involve gaining access to information on the physical and mental constitution of an individual without their knowledge or consent. Such breaches are becoming increasingly likely in an era marked by massive health-based data collection and digitization efforts, whose ultimate goals include the provision of personalized medical interventions. Genetic data lie at the heart of these tailored medical approaches, as many phenotypes are believed to have an ultimate basis in our genetic makeup, and as such, are being collected as a part of large-scale projects such as the UK Biobank (https://www.ukbiobank.ac.uk) and the National Institute of Health (NIH)'s All of Us (https://allofus.nih.gov) program.

Pioneering work by Homer et al. (2008) showed that genetic data in general, and single nucleotide polymorphisms (SNPs) in particular, enable the identification of individuals in DNA mixtures. Additional work reaffirmed the ability of SNPs to reveal whether an individual belonged to a study cohort or DNA mixture (Visscher and Hill 2009). Gymrek et al. (2013) exploited the possibility of linking the genomes of individuals to surname data from genealogy websites, thereby clearly demonstrating the risk of exposure from the public release of genotype data. Nowadays, law enforcement agencies frequently use SNP data in the identification of individuals, and their genetic relatives. Although it has been suggested 20–30 independent SNPs are enough to reidentify individuals (Lin et al. 2004), this quantification needs to be updated based on available databases of human genomes and auxiliary biological data.

SNPs can now be easily and cheaply extracted from genotyping and omics assays, say, in genetic studies of disease vulnerability; forensics analyses of found objects; and can be inferred from established genotype-phenotype relationships. Functional genomics assays allow both direct sequence-based variant discovery (Gürsoy et al. 2020), and inference using gene expression values and associated loci (Harmanci and Gerstein 2016). The ubiquity of large-scale omics projects make this source of variants especially concerning (Gürsoy et al. 2020). Several studies have showed identification risk even with partial privacy preservation measures such as SNP beacons (Shringarpure and Bustamante 2015; Raisaro et al. 2017; Von Thenen et al. 2019), and the publication of Genome wide association studies (GWAS) summary statistics (Im et al. 2012). For some time, there has been a strong case for the restricting access to genotypic data and comprehensive privacy preservation, say, through encryption-based analysis (Erlich and Narayanan 2014); however, for genotypes inferred from functional genomics data or environmental samples, the field is now directing attention towards notions of data sanitization (Gürsoy et al. 2020).

Arguments for greater protections are often confronted by the biomedical community's desire for unrestricted access to data sets: increased public access would democratize information, enabling biological analyses of greater statistical power in proportion to data size and quality. Striking a balance requires clear reidentification and inference risk quantification, relative to the proposed benefit of data release. In response to this, we provide a computational tool that assesses the degree to which a set of released SNPs could lead to genotypic and phenotypic inferences, using a hidden Markov model (HMM) approach (Fig. 1A,B). The tool is termed “Privacy Leakage by Inference across Genotypic HMM Trajectories” or PLIGHT.

**Figure 1.**
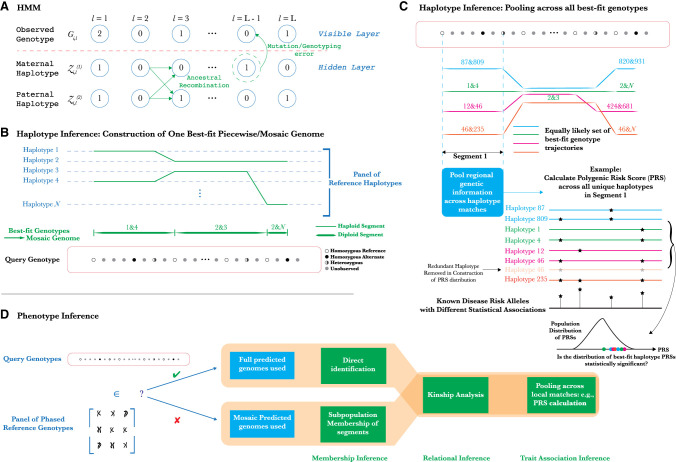
Methodology of PLIGHT's HMM-based inference and downstream analyses. (*A*) Schematic of the Li-Stephens HMM for haplotype recombination and mutation. (*B*) Inference of best-fit haplotype pairs in different genomic regions based on a chosen database of reference haplotypes, and the subsequent construction of the diploid mosaic genome. (*C*) An example of how the haplotype inference procedure described here could be used for novel attacks. By calculating polygenic risk scores (PRSs) for traits across all identified mosaic genome segments, we can test for statistically significant clustering of the PRSs relative to background values. If significant, the individual is more likely to be linked to such a trait. (*D*) Exploration of potential phenotypic inference attacks, conditional on whether or not the individual is known to be in the reference haplotype database.

The premise of PLIGHT is that even error-prone and sparsely distributed genotype data, especially SNPs, carry a risk of identification and downstream inference. We show here that it is often possible to expand upon the partial information from noisy and sparse SNP sets using existing genetic databases. For example, consider the case in which a single DNA fragment can be separated from contaminated genetic material, thus reliably providing information on a single source individual. With the advent of long-read technologies such as nanopore (Jain et al. 2016) and SMRT (Eid et al. 2009) sequencing, we can now read that fragment to a length of hundreds or thousands of base pairs. The data is somewhat error-prone because of the dearth of material, but still could yield a few noisy SNPs along the fragment. Additionally, certain physical characteristics, and knowledge of Mendelian disorders in individuals, potentially leak information on mutations at particular loci (Posey et al. 2019). Given the combination of population-scale public genotype databases and SNP sets associated with potentially socially compromising disease risk, assessing the identification risk for sparse SNP data is especially important.

Inspired by imputation methods such as IMPUTE2 (Howie et al. 2009) and Eagle (Loh et al. 2016), PLIGHT's inference procedure is based on the Li-Stephens model (Li and Stephens 2003), where an HMM is used to explore the space of underlying pairs of haplotypes in a diploid genome with possible de novo mutations and recombination between haplotypes. In contrast to imputation, however, PLIGHT does not identify the most likely SNPs at each genomic locus (which would be an under-constrained problem for very sparse SNP sets). Rather, a solution to the inference problem consists of finding which segments of existing haplotypes in a database best match the query SNP set. These segments are pieced together to form a best-fit continuous path, or genotypic *trajectory*, through the reference haplotype space. Thus, whereas phasing and imputation already have highly efficient solutions to find the single most likely genotype at an unobserved locus based on sufficiently dense nearby SNPs, our method is optimized to find all equally likely trajectories pieced together from reference haplotypes.

Several studies of genetic privacy have considered *k*th-order correlations between SNPs (*k* = 2 corresponds to pairwise linkage disequilibrium [LD]) to maximize the probability of reidentifying individuals (Wang et al. 2009; Samani et al. 2015; Von Thenen et al. 2019). These studies showed the increased ability to identify individuals when SNP correlations are exploited. PLIGHT seeks to capture all these higher order correlations, using the preexisting correlation structure (of *any* order) of the reference genetic database with a biologically plausible model of recombination (the default is based on HapMap Consortium data [The International HapMap Consortium 2003; Li and Stephens 2003; Marchini and Howie 2010], though users can add custom, position-specific recombination rates). In contrast, assuming independent SNPs for inference requires the quantification of genotype probabilities. This, in turn, necessitates a reference population to be defined for the allele and genotype frequencies to be calculated. In large databases, using average frequencies across reference individuals with mixed local ancestries may reduce the ability to find individuals. PLIGHT is agnostic to any assumptions of subpopulation membership of the query individual, population homogeneity or to estimates of allele frequencies; rather, it is dependent on the composition of the reference database. Additionally, we leverage the inference procedure to make a more informed choice of which SNPs to sanitize in data sets whose primary purpose is not the identification of variants (e.g., RNA-seq or ChIP-seq data). We include the sanitization tool in the PLIGHT suite.

The employment of HMMs to model LD in genomes has substantial precedent, such as in local ancestry inference (Pasaniuc et al. 2009; Price et al. 2009; Baran et al. 2012) and the determination of Identical-by-Descent (IBD) genomic regions (Bercovici et al. 2010). For example, Baran et al. (2012) use a latent-state-space reduction approach by running a two-level HMM: an inner model for each ancestral population in a genomic window of a certain length and a higher-level model for exchanges between windows. We choose a more straightforward implementation of HMMs, to avoid assumptions either on the density of query SNPs (defining windows precludes recombination between SNPs in that window) or of ancestral membership. There is, however, a consequent price paid in an increase in running time and memory usage. We partially ameliorate these costs using sampling and pooling approaches. We also note that the positional Burrows–Wheeler transform (PBWT) (Durbin 2014) has been used in tandem with the Li-Stephens model to improve the efficiency of haplotype matches across large databases (*fastLS* [Lunter 2019]). The scaling of this method with database size is far superior to a straightforward HMM implementation. However, for now, *fastLS* does not allow position-specific variations in the recombination rate. For sparsely distributed SNPs, there will be large variability in the recombination rates between any pair of adjacent SNP sites. PLIGHT allows for variation of the recombination model, and even position-dependent recombination effects. Further, in contrast to the PBWT formalism, PLIGHT also enables the user to include trajectories that are less-than-optimal, for robustness checks or exploration of noisy data.

The primary aims of this study are: (1) To show that even a few SNPs leak considerable information about source individuals, allowing them and genetic relatives to be discovered in databases, and permitting matches of subsegments of their genotypes to reference databases; (2) To provide tools to quantify information leakage and to subsequently sanitize query SNPs in a manner that balances utility of data sets and privacy of the source individuals.

## Results

### Repurposing population-genetics models for privacy

The primary purpose of PLIGHT is to quantify SNP information content, and to leverage that information to make judicious choices on SNP masking (*sanitization*) from data sets. The expectation is that an attacker would acquire a set of unphased alternate allele dosages {0, 1, 2} at SNP loci. Inference using a reference genetic database requires that SNP positions overlap, at least partially, those genotyped in the database. Each identified SNP may have a unique probability associated with the alternate allele dosage (reflecting certainty) or an overall error rate may be assigned. The Li-Stephens HMM (Fig. 1A) then maps recombination and mutation processes onto the transition and emission probabilities, respectively. Thus, query genotype alignment can be seen as simultaneously phasing the genotype into the paternal and maternal haplotypes at a single query locus, and allowing for transitions to other haplotypes between adjacent query loci. In doing so, segments of the query SNPs are found to match to pairs of haplotypes which may transition to other pairs for the next segment (Fig. 1B).

Formally, we find the trajectories as sequences of reference haplotype pairs (for a diploid genome) at each locus that best fit the observations: that is, for observed query SNPs at genomic loci *l* = {1, 2,…, *L*}, the trajectory $T=\;{\left\{\;j(l),k(l)\right\}}_{l=1}^{L}$, where *j* and *k* are the labels of best-fit reference haplotypes as a function of the observed genomic locus (Supplemental Fig. S1). We label a single best-fit trajectory as a diploid *mosaic* genome if the pairs of matching reference haplotypes change across the observed loci. The collection of all equally likely trajectories for a given query SNP set is the primary output of the algorithms.

Having access to all equally likely trajectories may enable novel forms of attack (Fig. 1C,D), which we explore below. PLIGHT also provides a visualization of all trajectories across the observed loci, and the logarithms of the joint probabilities of observing the query SNPs for: (a) the HMM, and models where (b) SNPs are independent and satisfy Hardy-Weinberg equilibrium as defined by their minor allele frequencies (MAFs); and (c) SNPs are independent, but described by their genotype frequencies (not their MAFs). The log probabilities of the most likely trajectories are also presented. Data publishers have the option of removing (“sanitizing”) the most informative SNP from the observed sample, based on identifying segments with the smallest diversity of inferred trajectories (i.e., segments with the fewest trajectories passing through them) and the SNP allele frequencies.

A major design consideration was contending with reference database size: with the search space ranging from thousands to (eventually) hundreds of thousands of genotypes, HMMs quickly become computationally intractable. Our approach was to construct three algorithms that negotiate the tradeoff between exactness of the calculation and the memory burden:

1. *PLIGHT_Exact* performs the exact HMM inference process using the Viterbi algorithm (Viterbi 1967); *PLIGHT_InRef* is the same algorithm with the recombination set to 0, for cases in which the query individual is known to be in a database.
2. *PLIGHT_Truncated* truncates the set of calculated trajectories to only those within a certain probability distance from the maximally optimal ones, resulting in a smaller memory footprint.
3. *PLIGHT_Iterative* iteratively partitions the reference search space into more manageable blocks of haplotypes and runs *PLIGHT_Exact* on each block, followed by pooling and repetition of the scheme on the resulting, smaller cohort of haplotypes. This algorithm has significantly better reference-database-scaling properties than the others.

*PLIGHT_Truncated* and *PLIGHT_Iterative* are approximations designed to reduce hard disk memory usage, and both hard disk and RAM usage, respectively. However, for most purposes *PLIGHT_Truncated* is superseded by *PLIGHT_Iterative* in performance. *PLIGHT_Truncated* mainly serves to determine the “compressibility” of the trajectories, that is, the size of the haplotype subspace that is sufficient to match the results of the exact algorithm.

**Figure 2.**
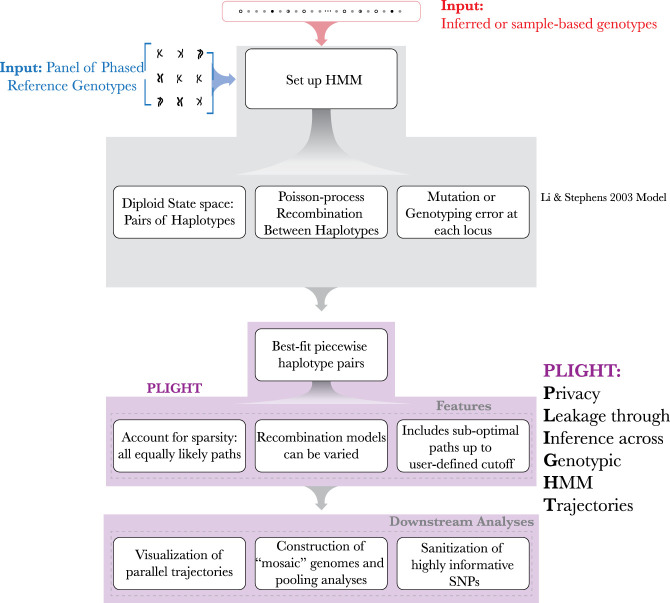
Computational framework of PLIGHT. The inputs of a reference database and of the query SNPs are shown at the *top*. Next, the population-genetics model by Li and Stephens forms the core of the HMM-based framework. This includes the definition of a diploid state space, a default Poisson process–based recombination model with growth rate proportional to the genomic distance between two loci, and a mutation/error rate at each locus. This model is implemented within PLIGHT, which identifies the best-fit reference haplotype labels in the diploid state space. Special features include the identification of all equally likely trajectories, flexibility in the recombination models used, and an allowance for sub-optimal trajectories to be identified. Furthermore, PLIGHT includes visualization and SNP sanitization modules. The framework also allows for the construction of mosaic genomes and analyses involving the pooling of information across all identified mosaic trajectories.

Subsequently, the results are visualized using *PLIGHT_Vis*, and processed using downstream modules. The user may use *PLIGHT_SanitizeGenotypes* based on these results: a SNP is eliminated from the query set, which is then rerun through one of the three HMM algorithms to produce the sanitized results. The overall computational framework is depicted in Figure 2.

### Attack scenarios

We outline examples of real-world scenarios in which we reasonably expect to observe a small number of noisy SNPs, the types of attacks that may be performed using those SNPs (Fig. 1D), and the applicability of PLIGHT to such attacks. One common element of these SNP sources is the likelihood of producing noisy SNPs, with differing degrees of inter-SNP correlation.

#### Sources of small numbers of SNPs and their characteristics

1. Low quality and /or contaminated samples: DNA samples acquired from contacted objects (such as coffee cups) can yield thousands of SNPs (e.g., Gürsoy et al. 2020). However, if DNA samples become damaged or contaminated by other DNA sources, the yield of high-confidence SNP calls may be significantly reduced (Tillmar et al. 2019; Turchi et al. 2019; de Vries et al. 2022). It is important to explore the lower bounds of SNP numbers that would suffice for certain types of privacy attacks, given the ease and legality of surreptitiously extracting variants from environmental DNA samples.
2. Nanopore reads: For samples containing DNA from multiple individuals, nanopore sequencing technologies could be used to read individual DNA strands. A single read from nanopore sequencing would reliably belong to a single individual (thus removing the effects of contamination), say, if it was drawn from a forensic sample. This would yield a handful of noisy SNPs, with a high degree of correlation because of genomic proximity.
3. Inferred SNPs: Although more speculative, we consider the possibility of sourcing SNPs from knowledge of individual characteristics. If even a few SNPs can be guessed, say, based on known Mendelian disorders in an individual or their genetic relatives, it might be possible to piece together sufficient genetic information to find such individuals (or relatives) in sensitive phenotypic study databases. Inference of SNPs has also been shown for derived genomic data, such as expression quantitative trait loci (eQTLs) (Harmanci and Gerstein 2016) and allele-specific expression (Gürsoy et al. 2021). Again, the number of inferred SNPs may be considerable, but with varying degrees of confidence. The highest quality matches would form a smaller subset. Finally, a small number of germline SNPs may be leaked in somatic variant call sets (Sendorek et al. 2018; Meyerson et al. 2020), though evidence is ambiguous as to the number of true germline variants.

#### Types of privacy attacks considered

1. Membership inference attack: The identification of an individual as a member of a database or study could allow the linking of that individual to potentially stigmatizing phenotypes. We consider two forms of membership inference:
  - *Full membership:* This is the case when an individual is in a database.
  - *Partial membership:* This is the case when partial segments of the query individual's genome are found in a database. This could occur because of IBD or Identity-by-State (IBS). Partial membership allows certain regions of the genome to be related to a phenotypic cohort; for example, the individual may overlap in certain genomic regions exclusively with individuals in the phenotype-positive group (i.e., with cases, not controls).
2. Relational (or Kinship) inference attack: This type of attack technically overlaps with the partial membership attack mentioned above, but warrants its own discussion. Partial matches in certain genomic regions could allow the genetic relatives of a query individual to be found in phenotypic databases, thus exposing them to privacy attacks.
3. Trait association inference attack: This involves using the partial information from regional matches to the genome to make inferences about the individual. Consider the following hypothetical example. An individual is in contact with an object from which the attacker collects DNA, identifies the subset of high-quality SNPs, and infers individual characteristics—such as the genetic subpopulation to which the individual belongs, or GWAS SNPs or PRSs for certain phenotypes.

#### Deploying PLIGHT to address attacks

We use unphased SNPs with a user-defined level of confidence/error rate as inputs. We show full membership attacks (a) in the presence of genotyping error and (b) when SNPs from other individuals contaminate a primary source. The full membership attack is the simplest form of HMM inference because we restrict the recombination rate to be 0 (and so the transition matrix is the identity matrix). This simple HMM is still different from the assumption of independent SNPs because potential correlations (LD) exist between query SNPs across the genetic database, and the model can account for errors.

We then search for relatives of query individuals, by combining partial genome matches across chromosomes. Finally, we explore a version of the trait association attack in which we use the matched segments (for individuals not in the database) to partially impute SNP dosages at GWAS SNP sites and calculate coarse-grained polygenic risk scores (PRSs; Fig. 1C). In this way, we chart out a comprehensive exploration of how to quantify risks, and end with a mechanism to carefully sanitize data sets.

The advantage of our approach over the simpler assumption of independent query SNPs is derived from the fact that SNPs are frequently correlated, either through random choice (see Supplemental Materials for an analysis on the random sampling of SNPs across a chromosome) or by design, or their correlation structure is unknown (say, if an individual's local ancestry varies in a manner hard to capture with simplistic boundaries between populations).

### Data sets and shared parameters

In following sections, we test our tools using a series of simulations. The primary reference set used is the 1000 Genomes Phase 3 database (The 1000 Genomes Project Consortium 2015) (based on human genome reference build GRCh37, FASTA file *human_g1k_v37.fasta* [ftp.1000genomes.ebi.ac.uk/vol1/ftp/technical/reference]), with 2504 phased genotypes in total from 26 different sampled populations. The methods rely on the availability of phased reference genotypes, read in as chromosome-separated variant call format (VCF) files. We emphasize that, whereas we used the Phase 3 database based on the GRCh37 genome, the same methods would carry over identically to GRCh38 or any other genome build. The only requirement is that the SNP positions in the query match up with those in the reference database. For all analyses, we filter out low allele frequency SNPs, with MAF restricted as: 0.05 ≤ *MAF* ≤ 0.5. We choose this range to quantify the leakage associated with even relatively common SNPs, as the identifying information in low MAF SNPs will be significant.

### Identification of individuals known to be within a database

We consider the simplest scenario, in which an individual is known to be in a database (using *PLIGHT_InRef*). We test examples with different numbers of SNPs and varying mutation/genotyping error rates, λ. For each of 5 λ rates, we run 10 iterations of the following: a single individual is randomly selected from the 1000 Genomes Phase 3 cohort of 2504 individuals, and the query genotype set is simulated (see Methods). The chosen individual is likely to be different for the 10 iterations (even though sampling was performed with replacement). We consider only one chromosome (Chromosome 17 was chosen at random) for this analysis.

For each error rate and iteration, we choose a single individual and simulate all 40 possibilities of *N*_*SNP*_ ∈ [1,…, 40] for that individual. The SNP selection is repeated for each of the 40 cases, so the SNP sets are not the same for the different values of *N*_*SNP*_. We simulate cases of homozygous or heterozygous alternate alleles, to mimic those reported from either SNP beacons or noisy functional genomics data. However, the inference would proceed identically if we included homozygous reference alleles.

We report the mean and standard deviation, across the 10 iterations, of the minimum *N*_*SNP*_ value for which the individual was uniquely identified (Table 1). With nonzero mutation rates, it is possible to erroneously find individuals different from the ground-truth individuals. We therefore also provide the mean and standard deviation of the minimum *N*_*SNP*_ for which a *unique and correct* identification is made. For reference, we also provide the mean and standard deviation of the pooled list of MAFs across all 10 iterations for each mutation rate.

**Table 1.**
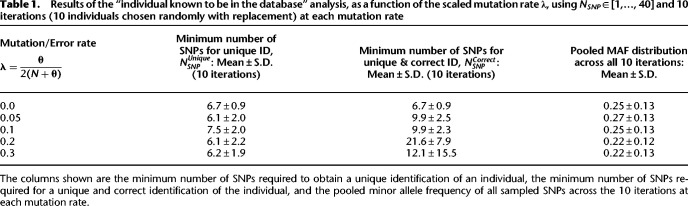
Results of the “individual known to be in the database” analysis, as a function of the scaled mutation rate **λ**, using *N*_*SNP*_ ∈ [1,…, 40] and 10 iterations (10 individuals chosen randomly with replacement) at each mutation rate

The results indicate that, whereas an identification can be unique for nearly the same average $N_{SNP}^{Unique}$ across all mutation rates, the ability to find the correct individual worsens with an increasing mutation rate. The mean value of $N_{SNP}^{Correct}$ does not increase monotonically, but the standard deviation does. We investigated this through an independent simulation, and found that this is likely because of poorer sampling at higher mutation rates: because $N_{SNP}^{Correct}$ is based on the number of correct and unique identifications of the query individual, if few or no such identifications are made at higher mutation rates, the statistics of $N_{SNP}^{Correct}$ will necessarily be impacted (Supplemental Results; Supplemental Fig. S2). It is still noteworthy that unique identification can be made with a very small number of common SNPs (∼6–8), with MAF values distributed fairly evenly across the range 0.05 ≤ *MAF* ≤ 0.5. Even in the presence of modest mutation rates (≲0.1), correct identification requires only about 10 common SNPs on average.

At the same time, this result may not be entirely surprising considering the number of markers used in forensics studies, as well as the information content of a SNP set under Hardy-Weinberg equilibrium (HWE). With respect to the former, we note that the CODIS database expanded the number of core loci used from an original 13 to 20 in 2017 (https://www.fbi.gov/services/laboratory/biometric-analysis/codis [accessed October 22, 2021]). Concerning the latter, it can be shown that for independent SNPs under HWE and with similar MAFs, a similar number of SNPs suffice to identify an individual in a database of the scale of the 1000 Genomes cohort (Lin et al. 2004). Our purpose here is to confirm the power of a small set of SNPs, and to show that PLIGHT can evaluate the identifiability of an individual irrespective of the LD structure and MAFs of the query SNPs, and even in the presence of moderate amounts of noise. Further, in cases in which SNP frequencies are reasonably correlated with each other, we expect PLIGHT to improve performance over a model assuming HWE.

### Identification of individuals from contaminated samples

To quantify the reidentification risk from samples that may have been in contact with multiple individuals, we run a simulated experiment. We (a) select five individuals; (b) draw 40 SNPs from a single chromosome (the “base” SNP set); (c) designate one individual as the primary “target” of the attack; (d) with a varying probability (ranging from 0 to 0.7), randomly replace the genotype dosage of each SNP of the target with a dosage from one of the other four individuals (with equal probability = 0.25); (e) starting with this “contaminated” SNP set, iteratively remove one SNP and run the inference procedure to determine if we can uniquely and correctly identify the target; (f) record the minimum number of SNPs allowing unique and correct identification. We run three iterations over the choice of individuals and the base SNP set, and 10 iterations in which the contaminated set is randomly generated anew from each base set. *PLIGHT_InRef* is used for inference. The replacement rate was also used as the error rate (λ) in the inference process (this is prior knowledge; if unknown, the user could try several possible error rates). To explore the impact of SNP diversity, we consider two cases for selecting individuals: the target and contaminant individuals are chosen from the full 1000 Genomes population (1kG), resulting in more diverse variation; or they are chosen from the CDX subpopulation (*CDX*) only. The results are in Table 2.

**Table 2.**
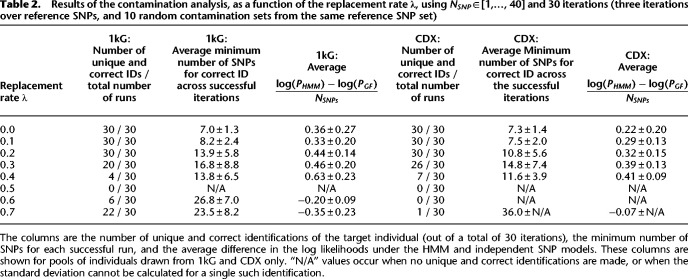
Results of the contamination analysis, as a function of the replacement rate **λ**, using *N*_*SNP*_ ∈ [1,…, 40] and 30 iterations (three iterations over reference SNPs, and 10 random contamination sets from the same reference SNP set)

We first note the expected behavior that, with increasing replacement rates, the average minimum number of SNPs for correct identification of the target increase. However, we also find an interesting trend for the 1kG pool, in which the number of successful identifications drops to 0, before increasing again at higher error rates. We do not see a similar trend with the CDX population. Further investigation (Supplemental Results) indicates that this results from setting the error rate to the replacement rate λ: whereas greater replacements of the query's genotypes make that individual harder to find, larger model error rates allow greater leeway in genotype matching. In particular, at an error rate of 0.5, the conditional probabilities for all observed genotypes are equal for all combinations of the reference haplotypes (Supplemental Table S1). This means that all combinations of reference haplotypes are equally likely and no unique identification of the query individual is found. Rerunning the inference in independent simulations with an error rate of 0 (Supplemental Results; Supplemental Fig. S3) leads to these overall conclusions: Unique and correct inference becomes more difficult at higher replacement rates, as expected; allowing the error rate to vary helps with inference when there is uncertainty about sample purity.

We used PLIGHT's output of various probabilities to determine the degree to which the SNPs were independent. Here, we evaluated the per-SNP difference between the logarithm of the joint probability of SNPs under the HMM (log(*P*_*HMM*_)), and under the independent SNP model. For the latter, we considered genotype frequencies (log(*P*_*GF*_)) rather than MAFs under Hardy-Weinberg equilibrium (though both are output by PLIGHT) as *PLIGHT_InRef* focuses on matching genotypes and not haplotypes. We see that the log(*P*_*HMM*_) > log(*P*_*GF*_) on average for low mutation rates, but this trend reverses at higher mutation rates. At low mutation rates, the nonindependence of SNPs would lead to a higher log(*P*_*HMM*_), whereas at high mutation rates the emission probabilities deviate sufficiently from 1 to lower log(*P*_*HMM*_) relative to log(*P*_*GF*_). For a small number of runs, a small negative $\frac{\log(P_{HMM})-\log(P_{GF})}{N_{SNPs}}$ was observed even with 0 replacement rate (Supplemental File S1). This is likely because of round-off errors.

### Mosaic overlap between query individuals and database individuals

We next evaluated the performance of PLIGHT on “mosaic” individuals, that is, individuals whose genome is constructed by sampling the diploid genomes of two or more source individuals, using simulated analyses. A pair of individuals is chosen at random from the 1000 Genomes cohort. The diploid mosaic genome is simulated for *N*_*Chr*_ sampled chromosomes (details in Methods).

#### Exact search within a reference database of 400 haplotypes

Here we use *PLIGHT_Exact*, using the full Li-Stephens model. Two individuals were selected, and the first half of the SNP genotypes were taken from one individual and the other half from the second. The mutation rate was set to 0, whereas the fixed per-base recombination rate *c*_*l*_ was set at 0.5 cM/Mb and the default linear recombination model was used. *c*_*l*_ = 0.5 cM/Mb was chosen close to the value of 0.4 cM/Mb, a biologically plausible average rate of recombination used in previous HMM studies (Li and Stephens 2003; Howie et al. 2009). This value led to reasonable exploration of the haplotype space, without devolving into a uniform consideration of all haplotypes as equally probable. A very low value of *c*_*l*_ will prefer to elongate the same haplotypes across all observed SNP positions without crossing over, that is, the emission probabilities will dominate in the HMM likelihood; a very high value will lower the barrier to crossovers, allowing frequent jumps to other haplotypes, that is, the transition probabilities will dominate in the HMM likelihood.

To reduce the matrix dimensionality, we restrict the search to 200 reference individuals (=400 haplotypes). We sampled *N*_*SNP*_ = 30 from each of three chromosomes (Chromosomes 1, 2, and 21). The two sampled individuals were HG00360 (first half) and HG00342 (second half). Note that, given the SNP selection process for simulated queries, the total query range was much smaller than the length of the chromosomes: in this example, SNP positions ranged from 1.03 Mb to 37.3 Mb for Chromosome 1, from 0.4 Mb to 22.1 Mb for Chromosome 2, and from 14.7 Mb to 38.4 Mb for Chromosome 21. This would inflate the degree of SNP correlation over SNPs randomly sampled across the full length. However, this is precisely the situation in which we expect PLIGHT to differ from independent SNP models, and so use it for exploratory purposes.

**Figure 3.**
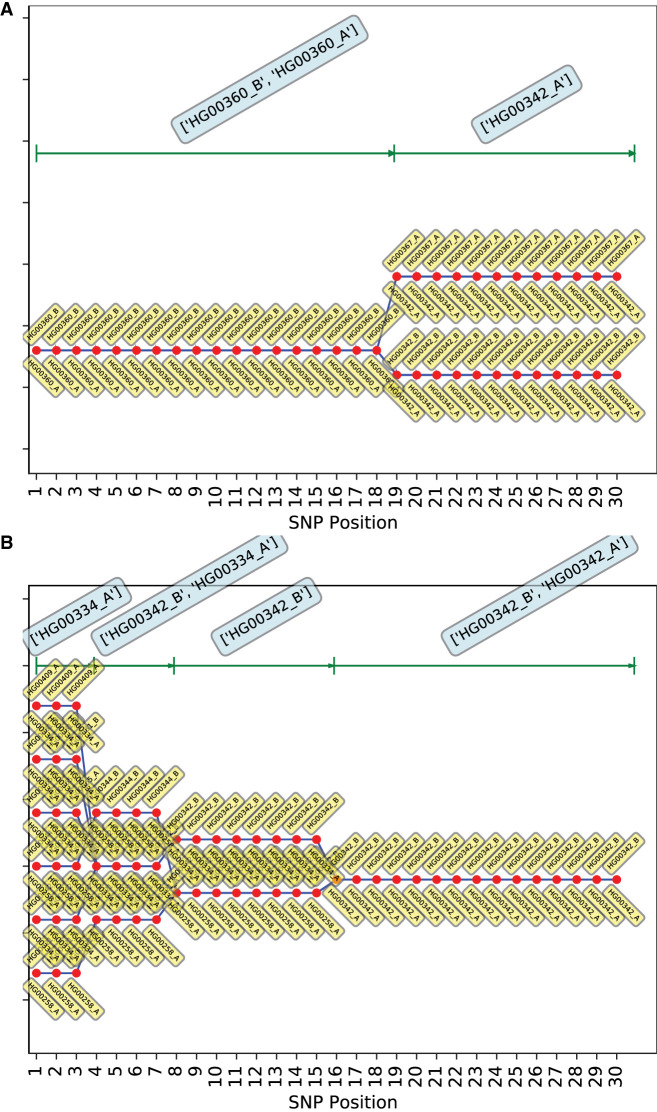
Best-fit genotypic trajectories from *PLIGHT_Exact* for the diploid mosaic genome of HG00360 + HG00342 constructed across 30 SNPs each for Chromosomes 1 and 2. The composition of the best-fit pair of haplotypes at each locus is depicted by two yellow tags, one *below* and one *above* the red dots. (*A*) Trajectories for Chromosome 1. (*B*) Trajectories for Chromosome 2.

Figures 3A and 3B show the trajectories for Chromosomes 1 and 2, respectively (Chromosome 21 results in Supplemental Fig. S4). Our labeling scheme involves splitting phased reference genotypes into the two parental haplotypes, with the arbitrary haplotype labels “A” and “B” appended to the reference individual ID. The labels for best-fit haplotype pairs are depicted by yellow tags below and above the red dot marking each locus of each trajectory. The results for Chromosome 1 indicate that the correct mosaic was identified as one of the two trajectories. The second trajectory consists of a mixture of one haplotype from the true individual (HG00342) and the other from a different individual (HG00367). The two trajectories branch out from HG00360 at the same SNP, indicating that for the last set of SNPs, HG00367_A and HG00342_B are likely identical (there is no noise in this simulation). The VCF files confirm the equality of genotypes for the last 13 SNPs. The haplotypes occurring with maximum frequency for certain stretches of the chromosome are identified at the top, with boundaries between the stretches marked by green ticks. Though the simulation drew the last 14 SNPs from HG00342, the transition from HG00360 to HG00342 in the solution occurs only for the last 12 SNPs because trajectories require a certain number of additional genotypic steps to build enough probability to warrant a transition. Thus, given the inference process is a combined approach to identify best-fit stretches of genotypes + boundaries between stretches, there is a certain fuzziness in boundary identification. The trajectories for Chromosome 2 (Fig. 3B), on the other hand, include HG00342, but not HG00360. Several alternate trajectories and branch points occur in the region of the true HG00360 segment. We speculated that, at the current recombination rate, it is more likely for one of the haplotypes (HG00342_B) to be extended across a longer stretch of the chromosome, which then results in alternative haplotypes being selected for the shorter remaining segments. To test this, we doubled the previous recombination rate (=1 cM/Mb). The results (Supplemental Fig. S5A–C) indicate that increasing the recombination rate does indeed help find the HG00360 segment. Higher recombination rates allow more haplotype transitions, leading to better exploration of the haplotype space. This is also evident from the increase in the average number of best-fit haplotypes per SNP. The results for Chromosome 21 (Supplemental Fig. S4) do include HG00360, in addition to several alternative trajectories.

#### Truncated algorithm

Running the same SNP set through *PLIGHT_Truncated* to assess the potential for memory reduction using fewer trajectories indicates considerable compressibility of the best-fit trajectories (Supplemental Results; Supplemental Fig. S6A–C).

#### Iterative and approximate search in a reference database of 5008 haplotypes

**Figure 4.**
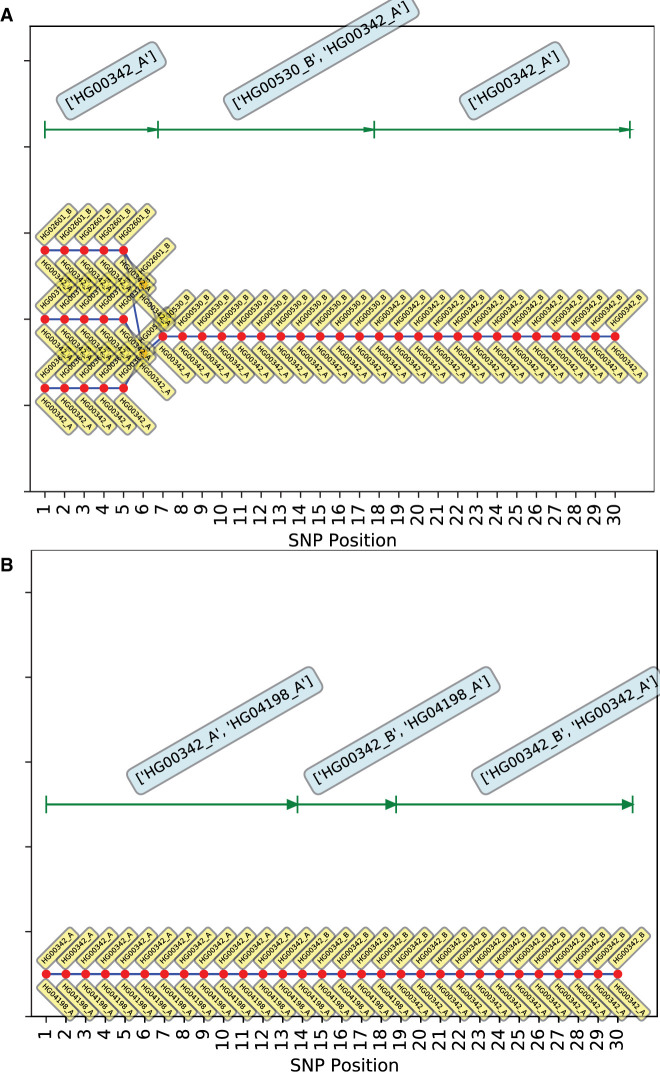
Consensus genotypic trajectories from *PLIGHT_Iterative* for the diploid mosaic genome of HG00360 + HG00342 across 30 SNPs in Chromosome 1, where the consensus score is evaluated by weighting the haplotypes in each trajectory in proportion to their occurrence across all three chromosomes. The composition of the best-fit pair of haplotypes at each locus is depicted by two yellow tags, one *below* and one *above* the red dots. (*A*) *n*_*iter*_ = 20, replicate 1; (*B*) *n*_*iter*_ = 30, replicate 1. Shown at the *top* of each panel are the most frequent haplotypes within each segment indicated. The second replicates of *n*_*iter*_ = 20 and *n*_*iter*_ = 30 are shown in the Supplemental Materials.

We run *PLIGHT_Iterative* on the same mosaic SNP set, but search through the entire 1000 Genomes database of 5008 haplotypes. We run two replicates for each of two sets of parameters: number of iterations (a) *n*_*iter*_ = 20; and (b) *n*_*iter*_ = 30; the subgroup size, *S*_*sg*_, is chosen to be *S*_*sg*_ = 300, and *c*_*l*_ = 0.5 cM/Mb. The stochasticity of the algorithm is apparent from the fact that there is no unequivocal improvement in the identification of component individuals from *n*_*iter*_ = 20 to *n*_*iter*_ = 30. Although it is possible that a significantly larger increase will improve mixing of the haplotypes, these runs are mostly successful at identifying the mosaic components, especially when information across chromosomes was combined. Accordingly, the default is set to *n*_*iter*_ = 20. The *consensus* trajectories, containing haplotypes frequently observed across chromosomes (see Methods), include both HG00342 and HG00360 in three out of four runs (though not necessarily within the same trajectory). Additionally, the query SNPs on each chromosome have different capacities for discerning the ground truth. The consensus results for Chromosomes 1 (one replicate with *n*_*iter*_ = 20 and *n*_*iter*_ = 30 in Figures 4A and 4B, respectively, and the other replicate in Supplemental Fig. S7A,B) and 21 (Supplemental Fig. S8A,B) include the true HG00360 + HG00342 combination within the same trajectory for one and two out of four runs, respectively.

Overall, we show that PLIGHT does not just allow reidentifying a specific individual in a database, but also finds individuals contributing to local genomic segments.

### Kinship analysis

Having analyzed simulated mosaics, we now benchmark the methods on known, *natural* mosaics in the form of a kinship analysis. We use a set of 13 related individuals (ftp://ftp.1000genomes.ebi.ac.uk/vol1/ftp/release/20130502/supporting/related_samples_vcf/related_samples_panel.20140910.ALL.panel) to link them to their first- (parents, children, or siblings), second- or third-order relatives among the 1000 Genomes Phase 3 main cohort of 2504 individuals (pedigree file: ftp://ftp.1000genomes.ebi.ac.uk/vol1/ftp/technical/working/20130606_sample_info/20130606_g1k.ped). For this study, we choose a single chromosome for each individual with the parameters: (I) *N*_*SNP*_ = 20, *n*_*iter*_ = 20; and (II) *N*_*SNP*_ = 30, *n*_*iter*_ = 30; the subgroup size was *S*_*sg*_ = 300, and *c*_*l*_ = 0.5 cM/Mb. The chromosomes were chosen at random, and are different in general between cases (I) and (II). A successful identification is indicated as the inclusion of the related individual anywhere within the best-fit trajectories. The results are presented in Table 3.

**Table 3.**
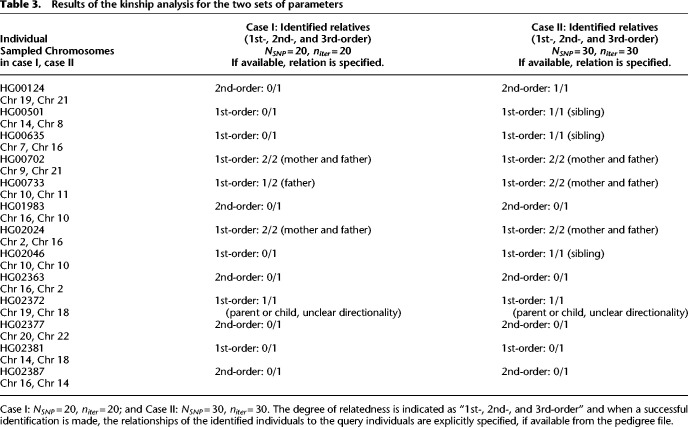
Results of the kinship analysis for the two sets of parameters

The results are impacted by several parameters: (a) *n*_*iter*_, (b) *N*_*SNP*_, and (c) chromosome identity. Based on the preceding section, it is likely that changing *n*_*iter*_ had minimal impact. Previous results also indicated that the choice and number of SNPs and chromosomes will impact the best-fit trajectories. We observe that irrespective of the query individual and chromosomal choice, increasing *N*_*SNP*_ clearly improves the efficiency of the kinship discovery (comparing Table 3, columns 2 and 3). Although no identification was made for certain individuals, there is an expected increase in risk for 1st-order relatives compared to 2nd-order relatives. A 2nd-order relative was discovered for a query individual only in one instance, where there were no 1st-order kin in the cohort to confound the identification.

However, even for 1st-order relatives, perfect identification was not possible. For example, the sibling of HG02381, present in the 1000 Genomes cohort, was not discovered even for *N*_*SNP*_ = 30. We subsequently used the 93 individuals belonging to the same population group as reference for a *PLIGHT_Exact* run. In this more restricted case, one of the haplotypes of the sibling was found as a match, but only for a part of the trajectory. The relative therefore remained concealed because of a combination of inherent SNP informativeness (evidenced by limited kin identification even on a smaller, exact run), database size, and algorithmic approximation.

We did not conduct a comprehensive search for the minimum number of SNPs to identify relatives because (a) *PLIGHT_Iterative* is computationally intensive and iterating over multiple query sets for many individuals can become unfeasible, and (b) without prior knowledge on which genomic regions overlap between relatives, changing the number of random SNPs may not yield improvements. In general, we show that even a modest number of common SNPs can reveal genetic relatives, especially 1st-order relatives, in a cohort of ∼5000 haplotypes.

### Inference based on SNPs obtained from environmental samples

As a real-world test, we considered SNP sets from DNA collected by swabbing glass slides containing dried saliva collected from two consenting individuals (Individuals A and B), with contamination simulated by preparing saliva mixtures at a 9:1 ratio (A:B, see Methods). This serves as a particularly challenging mimic of surreptitiously acquired DNA samples which pose significant identification risks to individuals. Our approach was inspired by our previous work in which swabs were taken from used, disposable coffee cup lids (Gürsoy et al. 2020). However, unlike the previous study in which samples were taken from lids used by a single person, here we deliberately introduced contamination from a second individual's saliva. This deliberate contamination is a controllable surrogate for environmental noise in the measured SNPs, and one in which appropriate consent from participating individuals was possible.

We randomly select 30 SNPs each from Chromosomes 3 (∼position 1 Mb to 190 Mb) and 6 (∼position 3 Mb to 169 Mb). We build references by combining blood-tissue-derived genotypes from Individual A as the gold-standard, and the 1000 Genomes genotypes. Twenty-nine SNPs out of 30 from both chromosomes overlap with the blood-tissue and 1000 Genomes VCFs (i.e., one query SNP from each chromosome is missing from the reference).

We check the 60 SNPs obtained from the environmental sample against the true genotypes of the query individual: six genotypes out of 30 for Chromosome 3 are incorrect, whereas four genotypes out of 30 are incorrect for Chromosome 6 (none of the incorrect SNPs correspond to the query SNPs that are missing from the reference). The incorrect calls are likely to arise because of possible contamination of the DNA of Individual 1 with that of Individual 2, as well as low sample read counts. Accordingly, we include nonzero mutation rates in our inference to account for errors or contamination. The environmental sample SNPs are unphased (because of noise and interindividual contamination). The gold-standard reference genotypes, from Individual A's blood tissue sample from Individual A, were phased (see Methods). We carry out two analyses, one with Individual A added to the 1000 Genomes reference database and the other with 1000 Genomes individuals only.

#### With 30 SNPs: query individual in the reference database

We merge the gold-standard SNP set with the 1000 Genomes VCF file, ensuring that only overlapping SNPs are retained. We determine whether 30 SNPs each on Chromosomes 3 and 6 would be sufficient to identify the true query individual, using both *PLIGHT_InRef* (i.e., *PLIGHT_Exact* with recombination rate = 0) and several runs of *PLIGHT_Iterative*. The *PLIGHT_InRef* algorithm, with a mutation rate λ = 0.1, correctly and uniquely identifies the query individual out of the full set of 2504 genotypes for both query chromosomes separately. We run four inference runs using *PLIGHT_Iterative*, where the full set of 5008 (1000 Genomes) + 2 (Individual A) haplotypes is used as the reference database: (a) *S*_*sg*_ = 200, mutation rate λ = 0.1; (b) *S*_*sg*_ = 200, λ = 0.2; (c) *S*_*sg*_ = 300, λ = 0.1; (d) *S*_*sg*_ = 300, λ = 0.2 (*n*_*iter*_ = 20 in all cases). The subgroup size *S*_*sg*_ is varied to examine the stability of results to different bootstrap sample sizes. For both Chromosome 3 and Chromosome 6, all the *PLIGHT_Iterative* runs yielded one of Individual A's haplotypes, whereas the second haplotype was not found. This is likely because errors in the environmental sample may have led to an alternate haplotype being selected from the 1000 Genomes database. Algorithmic stochasticity is apparent from slight variations in the best-fit trajectories.

#### With 30 SNPs: query individual not in the reference database, and coarse-grained imputation

The second analysis involves running *PLIGHT_Iterative* on the query SNPs to search through the reference database without including Individual A's genome. We run the same four combinations of parameters as before. For both chromosomes, we find certain haplotypes and haplotype combinations repeatedly in multiple runs, indicating that the algorithm finds close matches to the query SNPs. We use some of these best-fit trajectories to explore other instances of inference below.

We take trajectories from one run (*S*_*sg*_ = 200, λ = 0.2) and reconstruct the mosaic genomes. We use the overlapping SNPs between the gold-standard query VCF and the 1000 Genomes VCF to determine the total fraction of matching genotypes, and the correspondence score *C*, a weighted fraction that accounts for SNP rarity (see Methods). Background distributions are calculated from approximately 100 randomly chosen genomes from the 1000 Genomes reference, ensuring there is no overlap with mosaic genome haplotypes. There is one matching trajectory for Chromosome 3 and two trajectories for Chromosome 6. The exact-matching-genotype fraction for the trajectory in Chromosome 3 is 0.43 (background 0.36–0.45) [One-sample *t*-test *P*-value = 2 × 10^−16^]. For Chromosome 6, the matching fractions are 0.42 and 0.40 (background 0.35–0.45) (*P*-value = 0.21). The correspondence scores are: Chromosome 3, *C* = 0.33 (background 0.28–0.34) [*P*-value = 2 × 10^−16^]; Chromosome 6, *C* = 0.32, 0.32 (background 0.30–0.33) [*P*-value = 0.012]. Overall, although there is slight evidence for correct imputation of the intervening genomic regions between observed SNPs, we sought to more firmly establish the conclusions, because the mosaic scores still fall within the background ranges.

#### With 90 SNPs

We expanded the SNP set by selecting an additional 60 SNPs from Chromosomes 3 and 6. For Chromosome 3, 87 are found in the gold-standard blood-tissue genotypes, 90 in 1000 Genomes Phase 3; for Chromosome 6, 88 are in the gold-standard blood-tissue genotypes and all 90 in 1000 Genomes. We repeat the aforementioned tests for this extended set of ∼90 query SNPs. Running *PLIGHT_Iterative* on the in-reference case allows correct identification of both haplotypes of the query individual for all four runs for Chromosomes 3 and 6 (we do not run *PLIGHT_InRef*, as 30 SNPs already sufficed to find the query individual). Although some haplotypes occur in both the 30-SNP and 90-SNP runs, the resulting trajectories are different in general (i.e., different numbers and haplotype compositions).

We also use *PLIGHT_Iterative* without including the query individual in the reference. We use the run with *S*_*sg*_ = 200, λ = 0.1 to explore potential imputation. There are 10 best-fit trajectories for Chromosome 3 and 24 for Chromosome 6. The exact-matching-genotype fractions for the trajectories in Chromosome 3 are between 0.43–0.46 (background 0.36–0.44) [Welch's two-sample *t*-test *P*-value = 4 × 10^−8^]. For Chromosome 6, the matching fractions are between 0.39–0.42 (background 0.35–0.44) [*P*-value = 0.0003]. The correspondence scores are: Chromosome 3, *C* values between 0.33–0.34 (background 0.29–0.33) [*P*-value = 3 × 10^−11^]; Chromosome 6, *C* = 0.32–0.33 (background 0.30–0.34) [*P*-value = 7 × 10^−12^]. Overall, whereas the degree of matching improves slightly in going from 30 SNPs to 90 SNPs, the change is small. However, these results match genotypes across entire chromosomes; we hypothesize that within LD blocks associated with query SNPs we would observe better matching on average.

Thus, an individual can be robustly identified from amongst 5008 haplotypes using just 30 SNPs on a single chromosome, even in the presence of errors. Searching against a reference without the query individual yields robust best-fit trajectories found by *PLIGHT_Iterative* across different parameter settings. Expanding to a larger set of query SNPs indicates that the haplotype composition of the trajectories does change to reflect the new query SNPs, but with some haplotypes stably appearing in both sets of runs. The coarse-grained imputation at the level of 30 and 90 SNPs hints at only subtle differences relative to the background. We imagine that these differences would be amplified for a much larger SNP set, and when focusing on local regions of the genome in the vicinity of the query SNPs.

### Predicting genotypes at GWAS loci and polygenic risk score (PRS) analysis

Even partial, regional matches to query SNPs might allow inference of say, the risk of certain locus-specific genetic diseases. To evaluate this risk, we probe scenarios in which either excessive mutations or the absence of the query individual in the reference may prevent reidentification, but the set of inferred mosaic genomes may collectively provide hints about the query individual. We approximately calculate linear PRSs for the query and mosaic genomes for all phenotypes in the GWAS catalog version 1.0.2 (Buniello et al. 2019) to see if we could infer higher phenotypic risk for the inferred mosaic genomes relative to the background. We analyze the trajectories from a previous *PLIGHT_Iterative* run with *n*_*iter*_ = 20 and *S*_*sg*_ = 300, where the query individual was a composite of HG00360 (first half) and HG00342 (second half). There were 8, 15, and 2 trajectories for Chromosomes 1, 2, and 21, respectively. The PRS calculation was performed on all these trajectories independently and the PRSs across all trajectories (for each Chromosome separately) for each trait were compared with the PRSs for the mosaic as the ground truth. Four different statistical measures were evaluated (see Methods). This scenario is slightly artificial, as some of the best-fit trajectories for the HG00360-HG00342 example included the ground-truth individuals as well. Removal of these trajectories was not an option, given the pervasiveness of the query individuals in the results. However, we discuss this as an illustrative example of an aggregative attack. The results are in Supplemental Table S2. We also performed this analysis for the 30- and 90-SNP environmental sets (Supplemental Table S3). For the simulated and environmental sample PRS score comparisons, we mostly see a high cosine similarity between the true and mosaic reconstruction PRSs. However, the corresponding cosine similarities for the background individuals are similarly high. There is also noise in these metrics for all comparison cases, resulting in some negative or low cosine similarities. Therefore, whereas there are hints of some degree of aggregative leakage of information, the evidence in these examples is not very strong.

Instead, we sought to show the potential risks with an artificial example. We increased the likelihood of the query SNPs being in LD with the GWAS SNPs by choosing 90 SNPs, each within ±2 kb of known GWAS SNPs for the phenotype “Height.” We then evaluated both the degree of SNP imputation in the vicinity of the queries, as well as the inferred PRS scores, relative to a background population of 100 individuals (Supplemental Results; Supplemental Fig. S9). Neither test showed significant differences between the trajectory-based results and the background distribution.

Although not conclusive with the small numbers of SNPs considered here, our intention is to show the *types* of privacy incursions that may be conducted by utilizing partial inference. We foresee cases in which an attacker might apply such an approach to phenotypes such as eye color, which are associated with a relatively small number of SNPs (Meyer et al. 2020). The information leakage is likely to be stronger when many query SNPs are in strong LD with GWAS variants for the phenotype of concern. An attacker may query genomic regions proximal to disease loci, and even specifically amplify proximal genetic material.

### Sanitization of SNPs based on inferred trajectories

We provide a module to inform the removal of individual SNPs from a data set, called *PLIGHT_SanitizeGenotypes*. Sanitization would be applied to data sets whose primary purpose is different from the direct reporting of variants, but from which variants can be inferred. Functional genomics data sets are prime examples, as well as environmental samples in which the identity of any of the contributing individuals needs to be protected (see Supplemental Results for more examples). The algorithm is a modification of the simple strategy of removing SNPs based on rarity, that is, based on the MAF. The subset of SNPs with the fewest parallel best-fit trajectories are identified. These are “bottleneck” positions in the query set in which the number of parallel trajectories passing through them is the smallest, and so leak the maximum identifying information about the query individual. Specifically, the number of trajectories passing through a locus is quantified by the number of unique haplotype pairs (*N*_*UHP*_) at that locus (Supplemental Fig. S10; Methods). Often, multiple SNPs have the same number of trajectories passing through them, and so, of these, the SNP with the lowest MAF in this subset is removed. We emphasize that this is just one strategy among many possibilities, and depending on the use case, a user may make other sanitization decisions based on PLIGHT's output.

As a demonstration, we construct 10 mosaic SNP sets consisting of two source individuals, with half of the 30 SNPs drawn from one and the remaining from the other. We proceed to remove one SNP at a time, either based on the PLIGHT strategy or on the simpler MAF approach, and infer the best-fit trajectories on the new, sanitized SNP set. The results are compared to identify the relative merits of the two sanitization strategies.

The simplest way of assessing whether a sanitization strategy works is by calculating the degree to which the source individual is hidden among several others (the source individual will never be completely masked in a noise-free situation). Also, we can search for the source individual globally over the whole query set, or locally at particular sites. Accordingly, we settled on three metrics (definitions in Methods): (1) the total informational entropy of the identified individuals, *S*_*Ind*_; (2) the maximum probability of observing any of the two source individuals in the trajectory, $P_{Max}^{Source}$; and (3) the per-SNP entropy of identified individuals, if either of the source individuals is found at that SNP, *S*_*Per*−*SNP*_ (Supplemental Fig. S8). All three metrics are calculated as a function of the number of SNPs removed. For the ten examples, the number of SNPs removed is different as we stopped the removal process for each example once the number of inferred trajectories grew very large.

We find from the plots of *S*_*Ind*_ (Supplemental Fig. S11) and $P_{Max}^{Source}$ (Supplemental Fig. S12) that there is no pattern indicating that PLIGHT does a better job at increasing overall entropy, or that it reduces the probability of detecting the source individuals across all SNPs. Both these sets of plots evince considerable variability across runs. On the contrary, the plots of *S*_*Per*−*SNP*_(*i*) (Supplemental Figs. S13, S14) show a more consistent increase in the entropy per SNP for PLIGHT versus the MAF approach for those SNPs in which the source individuals are found: in Supplemental Figure S13, the overall distributions show greater per-SNP entropy for PLIGHT, whereas in Supplemental Figure S14, the entropy is generally higher for PLIGHT early on in the SNP removal process than for the MAF approach. This indicates that PLIGHT seems to improve the degree to which a source individual can be hidden at an individual SNP level, requiring fewer removals.

Of course, the main purpose of PLIGHT is not to determine an ideal method of data sanitization, but rather provide tools for users to choose sanitization procedures and numbers of SNPs to sanitize that best reflect their downstream publication goals. For example, consider a forensics study in which the data publisher intends to release a sample from a known individual, but hopes to protect the individual's relatives (who would only partially share genetic information). The target would be to hide the source individual's relatives (if known or published in other databases) among background haplotypes by removing SNPs that most strongly identify the relatives. The utility to be preserved would be the identifiability of the source individual. For a functional genomics data set, the utility to be preserved would be the genomic signal of interest, while hiding the source individual among background haplotypes (Gürsoy et al. 2020). A careful utility-versus-privacy analysis has to account for the intended data usage.

We also posit a possible interactive strategy for querying data sets, in which a data producer would allow an external user to query SNP genotypes from individuals in the “to-be-protected” data set. Often the data producer would like to enable a “locus-level” query on a genome (say, by visualizing in a browser) without compromising privacy. Here, “locus-level” refers to a genomic region, potentially containing several SNPs. The question then arises as to how many SNPs can be safely released. A more basic approach is to cap the number of SNPs at a fixed amount, but this does not take into account issues such as LD or higher-order correlations between groups of SNPs, and might be too restrictive. Our approach, on the other hand, would be the following. Based on the number and genomic positions of the SNPs, the data producer would run PLIGHT as a backend risk assessment tool to calculate the degree to which the source individual is hidden among background individuals. If the source individual is sufficiently obscured (say, is equally likely to be found as other background individuals), the data producer can release the queried SNPs—that is, the loci in a browser. Alternatively, the producer may prune any highly identifying SNPs and release a reduced version of the SNP genotypes to the user.

## Discussion

Our analyses reaffirm the prevailing notion that very few SNPs, even common ones, have the ability to leak identifying information about a query individual. Furthermore, we have shown that this leakage extends beyond the discovery of an individual known to be in a database, but can also provide piecewise genetic matches within databases, either exactly or approximately, conditional on any chosen recombination model. This idea of mosaic genotypic matching naturally includes the ability to identify genetic relatives within databases, and we show that our algorithm has the ability to discover 1st-order relatives such as parents, children, and siblings (and to a lesser degree, 2nd-order relatives) in cohorts of size ∼5000 haplotypes with as few as 30 common SNPs on a single chromosome. As shown in the contamination analyses, PLIGHT can find individuals even in the presence of significant amounts of contamination. Additionally, we show that the likelihood of observing some sets of SNPs (at lower contamination levels) under the HMM is higher than that under an independent SNP model. The upshot of these results is that investigators seeking to release genetic or omics data sets can use our tool to assess the degree to which to-be-published data could compromise the identity of a study cohort or related individuals.

It is our contention that this risk even extends beyond the identification of individual's or relatives. We have elucidated the capacity to extract further information from inferred trajectories. The process of identifying all genotypic trajectories that match a sparse set of data can be seen as a form of coarse-grained imputation: given a few SNPs, the genomic segments containing those SNPs are extended based on available reference haplotypes. However, instead of simply identifying one best-fit genotypic extension, we provide a larger list of all genotypic extensions consistent with the query set. In other words, we provide a sense of the “entropy” of the query genotypes conditional on a chosen reference, measuring the size of the best-fit genotypic state space in different genomic regions. An attacker could use the auxiliary information of individuals pooled across these regions to explore group phenotypic risk at particular loci, for example. We presented the results for one simplified case of such an attack, but a more sophisticated usage of the pooling attack could pick out risks for both Mendelian and complex genetic disorders.

We note that previous studies have used the Li-Stephens model to carry out genetic privacy attacks, albeit by imputing SNPs at unobserved sites. For example, (Samani et al. 2015) and (Raisaro et al. 2020) both use genetic imputation based on nearby genotyped variants to find the most likely SNPs at unobserved loci. Although these are powerful means of extracting further genetic information, for extremely sparse sets of SNPs, the use of conditional distributions of nearby SNPs will likely not be feasible. We therefore see PLIGHT as complementary to these approaches. We have endeavored to show that, even when exact imputation is difficult, leakage of information is possible. We foresee ways in which PLIGHT could fit into comprehensive privacy risk assessment platforms such as GenoShare (Raisaro et al. 2020) by adding complementary quantifications for sparse SNP data sets.

The expansion of genetic reference databases has also highlighted the limitations of the current reliance on single, linear reference genomes. Capturing the full range of genetic variation across a species (including SNPs, indels, structural variants [SVs], tandem repeats, etc.) in a manner that avoids biases associated with the choice of reference, requires data structures that are able to represent all possible sequence paths through available database genomes. Graph genomes (Novak et al. 2017; Paten et al. 2017) have been recently proposed as such frameworks for the generation of more inclusive reference databases, accounting for all known variants within a single traversable data structure. In one example (Rakocevic et al. 2019), sequences are represented as edges and breakpoints between variants as nodes. The number of branches extending out or into any node depends on the number of variants associated with that genetic locus. A single haplotype is constructed by starting at one end and traversing (in an acyclic manner) all branches that agree with the variants in the observed haplotype. If we now pose our query-matching problem in the context of graph genomes, we see that: (a) it is straightforward to locate the query genotypes in the matching graph edges, although we would have to include alternate branches weighted by the probability of de novo mutation or genotyping error; (b) identifying genotypic trajectories would amount to finding all possible paths that run through the branches determined in (a); and (c) it would be possible to account for linkage disequilibrium and the relative likelihood of each trajectory if the nodes of the graph genome were annotated with recombination rates calculated based on the reference database. In this way, we would be able to carry over the HMM approach into a graph genome context. If information on the number and identity of the reference haplotypes that map to each branch were also available, the pooling inference described above would also be possible.

Finally, there are further possibilities for using the underlying model for other problems, for example, the mapping of somatic mutations in cells to cancer lineages or viral mutations to clades. One interesting analysis would involve the iterative use of PLIGHT to parse through contributing genomes in a contaminated sample, by finding the most likely sets of SNPs arising from a single source genome. Ultimately, the generality and simplicity of the models make them attractive for multiple applications, as is clear from the ubiquity of HMM-based genomic analyses in the literature.

Our intention of quantifying privacy risks is, of course, complementary to subsequent sanitization procedures, such as those outlined previously (Gürsoy et al. 2020). In constructing *PLIGHT_SanitizeGenotypes*, we have envisioned one way of coupling the information from PLIGHT with data sanitization methods such as pBAM (Gürsoy et al. 2020) generation. By applying such an approach, we believe that a balance can be struck between the utility of the released data set and the associated privacy risk.

## Methods

### PLIGHT framework: Li-Stephens model and associated biological parameters

Let $G_{q}={\left\{G_{q,l}\right\}}_{l=1}^{L}$ be the genotypes of a query individual *q*, observed at SNP loci *l* = {1, 2,…, *L*}. The probability of observing such a genotype set given a space of reference haplotypes $H={\left\{Z_{\;j,l}\right\}}_{l=1;j=1}^{l=L_{Ref};j=N}$ (*L*_*Ref*_ = number of genotyped sites in the reference genomes, *N* = number of haplotypes in the reference database) is

(1) $$P(G_{q}|H)=\underset{Z_{j}^{\left(1\right)},Z_{k}^{\left(2\right)}}{\sum }P(G_{q}|Z_{j}^{\left(1\right)},Z_{k}^{\left(2\right)})\cdot P(Z_{j}^{\left(1\right)},Z_{k}^{\left(2\right)}|H),$$

where the set of all possible haplotypes at the observed loci on the two chromosomes is given by $Z_{j}^{\left(\alpha \right)}={\left\{Z_{\;j(l),l}^{\left(\alpha \right)}\right\}}_{l=1}^{L}$, $Z_{\;j(l),l}^{\left(\alpha =1,2\right)}$ is the haplotype at position *l*, and *j* is the index of the reference haplotype. The locus position is defined on a linear reference genome, with the genome build assumed to be the same between the query and reference database. We treat the haplotype index *j*(*l*) as a function of *l*, as it is possible for the fit reference haplotype to be different at each locus; that is, in the haplotype matching process, recombination between reference haplotypes may occur from one observed locus to the next (Supplemental Fig. S1). The second subscript explicitly indicates that, from haplotype *j*(*l*), we select the genotype at locus *l*.

We define a *trajectory* for a diploid query as $T=\;{\left\{\;j(l),k(l)\right\}}_{l=1}^{L}$, which is the sequence of reference haplotype pairs that best match the query SNPs. In the case of a haploid query, the trajectory would be a sequence of single labels at each locus.

The assumption of the observed genotypes and reference haplotypes being registered to the same, linear reference genome enables a simpler matching of reference haplotypes to observed genotypes. For data structures such as personal genomes and graph genomes additional genotype matching strategies would need to be incorporated, but the conceptual framework of searching through recombining haplotypes would be the same. In general, genotyped sites do not perfectly overlap with reference sites because of rare SNPs or differences in genotyping arrays. However, in our study we exclusively consider genotyped sites that overlap with reference sites, especially given our interest in common SNPs. If structural variants overlap SNP loci, we allow for missing values in the reference haplotypes (except for *PLIGHT_Truncated* because of methodological conflicts by including missing genotypes). We thus consider the reference haplotypes as providing the complete search space. We avoid making explicit assumptions of population membership and statistics for the query individual with the belief that, beyond implicit bias of a particular reference set, this will enable more unbiased, local estimates of kinship and genotypic similarity.

The two terms on the right-hand side of Eq. (1) are associated with an HMM (Li and Stephens 2003; Marchini et al. 2007): $P(Z_{j}^{\left(1\right)},Z_{k}^{\left(2\right)}|H)$ is related to the transition probabilities (and the interlocus recombination rate *c*_*l*_), and $P(G_{q}|Z_{j}^{\left(1\right)},Z_{k}^{\left(2\right)})$ to the emission probabilities (and the site-specific mutation/error rate λ, as shown in Supplemental Table S1). Details are provided in the Supplemental Materials.

### PLIGHT framework: hidden Markov model optimization

Given the characterization of the haplotype search problem as an HMM, identifying the best trajectory through haplotype space can be performed using the Viterbi algorithm (Viterbi 1967). This algorithm maximizes the trajectory probability in time *O*((*N* × *N*)^2^*L*), where *N* is the number of reference haplotypes, *N* × *N* is the corresponding number of diploid states, and *L* is the number of observed loci:

(2) $$\begin{array}{rl} Most\;likely\;trajectory & =\underset{Z_{j}^{\left(1\right)},Z_{k}^{\left(2\right)}\in {\left\{Z_{\;j(l),l}^{\left(1,2\right)}\right\}}_{l=1;j=1}^{l=L;j=N}}{\mathrm{argmax}}P(G_{q}|Z_{j}^{\left(1\right)},Z_{k}^{\left(2\right)})\cdot P(Z_{j}^{\left(1\right)},Z_{k}^{\left(2\right)}|H)\\ & =\underset{Z_{j}^{\left(1\right)},Z_{k}^{\left(2\right)}\in {\left\{Z_{\;j(l),l}^{\left(1,2\right)}\right\}}_{l=1;j=1}^{l=L;j=N}}{\mathrm{argmax}}P(G_{q},Z_{j}^{\left(1\right)},Z_{k}^{\left(2\right)}|H). \end{array}$$

Further details are in the Supplemental Materials.

A calculation of the *argmax* in Eq. (2) occurs separately at each pair of reference haplotypes and each locus, pointing to a set of best haplotype pairs at the previous locus. These sets of haplotype pairs must be stored in a “backtrace” vector. The maximum of this vector at the final locus then points back to the corresponding best reference haplotype pairs at the previous locus, and so on, resulting in a complete set of best-fit reference haplotypes at all observed query sites. However, the scaling of time complexity as *O*((*N* × *N*)^2^*L*) and of memory as *O*((*N* × *N*)*L*) strains computational resources. We thus modify the Viterbi algorithm to ameliorate these pressures on computing resources. First, we use the common logarithmic Viterbi algorithm to prevent round-off errors for vanishingly small probability products. Second, we incorporate aspects of the Li-Stephens model in a memory-efficient manner into the matrix evaluations (Supplemental Methods).

### PLIGHT framework: parallelization, truncation, and iterative schemes

We created three primary modules: First, the module *PLIGHT_Exact* encodes the full Li-Stephens HMM; second, the module *PLIGHT_Truncated* truncates the possible trajectory extensions at each observed site to a fraction of the total state space to reduce memory requirements; third, the module *PLIGHT_Iterative* slices up the total reference space into randomly chosen, tractable subsets, runs *PLIGHT_Exact* on the subsets, and pools the best states for a rerun. *PLIGHT_Truncated* was created to reduce the memory footprint on hard drives, as the backtrace vectors are memory-intensive. However, the amount of RAM consumed by *PLIGHT_Truncated* remains essentially the same as *PLIGHT_Exact*. The *PLIGHT_Iterative* algorithm was designed to ameliorate both the hard disk and RAM costs of the model, and is thus the preferred method for reference databases beyond ∼500 reference haplotypes. Below, we describe the parallelization procedure for speed-up, and additional steps taken in *PLIGHT_Iterative*, which are, in general, approximations of the full HMM. However, our results show that the approximations often approach the full model with judicious choices of parameters. Additionally, for *PLIGHT_Exact*, we explicitly design a variant module when the query individual is known to be in the reference database. This problem is one-dimensional in searching for genotypes versus the two-dimensional case of haplotype pairs, with the recombination rate = 0. This variant is called *PLIGHT_InRef*.

#### Parallelization scheme

In all modules, we use Python's *multiprocessing* scheme to parallelize calculations over all haplotype pairs. This greatly speeds up the analysis, as the matrix manipulations are some of the more time-consuming steps.

#### Truncation scheme

In *PLIGHT_Truncated*, at every observed site we truncate the possible haplotype states by choosing the top *T* sets of (α, β) pairs. This scheme was inspired by the Eagle2 imputation program (Loh et al. 2016). The premise is that, after a certain number of observed loci, only a fraction of the trajectories will meaningfully contribute to the best-fit states, reducing the storage of states in memory. Details are provided in the Supplemental Materials.

#### Iterative scheme

In *PLIGHT_Iterative*, the following steps are run:

- Set a tractable subgroup size *S*_*sg*_ for a single run of *PLIGHT_Exact*.
- Randomly shuffle the reference haplotypes, and chunk the full set into subgroups of size *S*_*sg*_.
- Run *PLIGHT_Exact* for each subgroup.
- Repeat steps (b) and (c) a user-defined *n*_*iter*_ times.
- Pool together all the best-fit haplotype pairs from all subgroups and iterations; best-fit pairs are separated into their constituent haplotypes at this stage.
- This pooled set is fed back into step (b) and the process is repeated until:
  - The length of the pooled list is smaller than *S*_*sg*_; or
  - The current pooled list is identical to the list derived during the previous pooling step; or
  - The current pooled list is larger than the previous pooled list.
- Once the outermost loop over pooling steps is exited, *PLIGHT_Exact* is run on the final best-fit list of haplotypes, with the output being a file with the best-fit pairs of haplotypes chosen from the final pooled list.
- The parallelization is run over the calculations in step (c).

The tradeoff here is, of course, between speed and memory usage. Searching through large databases could take significantly longer even when distributed in this manner, but the memory burden is substantially alleviated. An issue related to the subdivision process is that the globally optimal combinations of haplotypes, obtainable in an exact Li-Stephens HMM, may not co-occur in the subgroups defined here. This “mixing problem” is addressed in two ways: for the inner loop, we run the subdivision + *PLIGHT_Exact* process *n*_*iter*_ times, leading to *n*_*iter*_ × *n*_*subgroups*_ total runs, with *n*_*subgroups*_ = number of subgroups required to include all input haplotypes; for the outer loop, we obtain the pooled set of best-fit haplotypes across all *n*_*iter*_ × *n*_*subgroups*_ runs and input this union set to the next round of subdivisions. For the pooled set in the outer loop, we only include the individual haplotypes and not the haplotype pairs, as, if certain haplotypes have significant matches to the query, they will be retained through the different stages and allowed to combine with many other haplotypes. The loops are exited if there is no change in the set of best-fit haplotypes, or if the pooled set from one iteration is larger than that from the previous iteration (to prevent infinite loops), or if the pooled set can be easily run in *PLIGHT_Exact*. If the loop exits with a large number of best-fit haplotypes, we recommend rerunning the code (to allow the stochasticity to explore different trajectories) or modifying the parameters (e.g., the recombination rate or *n*_*iter*_).

### PLIGHT framework: visualization and analysis module

To visualize multiple trajectories with potential overlap, we constructed a processing and visualization module, termed *PLIGHT_Vis*. We build a graph with the best-fit haplotype pairs as nodes, the nodes being laid down in reverse order from the final observed site to the first. At every observed site, the union set of nodes (i.e., no repeats, even if the same node appears in several trajectories) are added and their connectivity with the soon-to-be-added layer at the previous site is established. This continues until the whole graph is constructed. The Python package Matplotlib is then used to generate a visual representation of the graph using linked lists. Arrows connect nodes (represented as dots) at one observed site to their corresponding descendants at the next site. The identities of the two inferred haplotypes at each node are printed above and below the dots.

Additionally, we provide two simple analysis tools:

1. For each chromosome, we present the maximally represented haplotype at each site at the top of the plot.
2. For cross-chromosome quantification of the most representative trajectories, we score each trajectory in each chromosome by a weighted sum,
  $$Score(tr)=\overset{H(tr)}{\underset{h=1}{\sum }}\overset{N_{chr}}{\underset{chr=1}{\sum }}P(h|chr)P(chr),$$
  where *H*(*tr*) = Number of unique haplotypes in trajectory *tr*, *P*(*h*|*chr*) = Probability of instances of haplotype *h* occurring in the predicted trajectory set for chromosome *chr*, *P*(*chr*) = Probability of *chr*, taken to be a uniform distribution $\Rightarrow P(chr)=\frac{1}{N_{chr}}$. *P*(*h*|*chr*) is calculated as the fraction of trajectories for *chr* within which the haplotype *h* occurs. The score of each trajectory is the sum of the cross-chromosome probability of a haplotype being found in the prediction, taken over all unique haplotypes in that trajectory. The cross-chromosome *consensus* trajectories with the maximum score in each chromosome are written to file. This weighting heuristic was chosen with the intention of identifying trajectories in each chromosome that share significant information with trajectories in all other chromosomes.

### PLIGHT framework: quantitative metrics

We output four quantitative metrics for the HMM modules. The first is the log-probability associated with the most likely trajectories, $\log P(G_{q},Z_{j}^{*(1)},Z_{k}^{*(2)}|H)$, where $\left\{Z_{j}^{*(1)},Z_{k}^{*(2)}\right\}$ represent the most likely trajectories. This metric is dependent on the number and MAF of the SNPs, as well as the degree of query matching to the reference haplotypes. Accordingly, this metric is not comparable across query SNP sets, but can be used to diagnose whether the same query set matches differently to a different database or with a different run of *PLIGHT_Iterative* (which has a certain degree of stochasticity).

The second metric is the logarithm of the joint likelihood of the SNPs conditional on the HMM:

(3) $$\log(P_{HMM})\equiv \log P(G_{q}={\left\{G_{q,l}\right\}}_{l=1}^{L}|H)$$

This is calculated as the total probability of the last step of the HMM, $P(G_{q}={\left\{G_{q,l}\right\}}_{l=1}^{L}|H)=\underset{Z_{j}^{\left(1\right)},Z_{k}^{\left(2\right)}}{\sum }P(G_{q},Z_{j}^{\left(1\right)},Z_{k}^{\left(2\right)}|H)$.

The third metric is the logarithm of the likelihood of observing the SNPs assuming they are all under Hardy-Weinberg equilibrium (HWE):

(4) $$\log(P_{HWE})\equiv \log\underset{l}{\prod }P_{HWE}(G_{q,l}|H),$$

where $P_{HWE}(G_{q,l}|H)=\left(\begin{array}{c} 2\\ G_{q,l} \end{array}\right){\left(MAF_{l}\right)}^{G_{q,l}}\cdot {\left(1-MAF_{l}\right)}^{2-G_{q,l}}.$

The final metric is the logarithm of the likelihood of observing the genotypes assuming independence, that is, instead of the alleles being under HWE, this just assumes that reference database captures the true independent probabilities of the diploid genotypes:

(5) $$\log(P_{GF})\equiv \log\underset{l}{\prod }P_{GF}(G_{q,l}|H),$$

where *P*_*GF*_(*G*_*q*,*l*_|*H*) = Frequency of occurrence of *G*_*q*,*l*_ in the reference population.

The log(*P*_*GF*_) metric is especially relevant for *PLIGHT_InRef*, where the genotypes are matched and not haplotype pairs.

### PLIGHT framework: sanitization module

We provide a simple tool for pruning a single SNP from the query set, based on the results of running PLIGHT. This tool, termed *PLIGHT_SanitizeGenotypes*, operates as follows:

1. The best-fit trajectories from a previous PLIGHT run are passed as input.
2. The SNPs for which the number of unique haplotype pairs (*N*_*UHP*_) is the least are identified (Supplemental Fig. S10). That is, for each SNP, the total number of unique haplotype pairs in the trajectories at that locus are counted, and those SNPs which have the smallest number are flagged. These subsets of query SNPs have the lowest “entropy” in terms of reference database matches, and are likely to contribute to easy reidentification. Formally, *N*_*UHP*_(*l*) = *No*. *of unique pairs of* {*j*(*l*), *k*(*l*)} *in the best* − *fit trajectories at locus l*.
3. We then rank these SNPs by MAF, and remove the one with the lowest MAF from the query list. A new, pruned query list is then generated and run through PLIGHT again.

This process can be repeated for every SNP a user chooses to remove.

The metrics considered for evaluation are:

1. Total informational entropy of the identified individuals: $S_{Ind}=-\sum_{k=1}^{N_{Ind}}P_{k}\cdot \log P_{k}$, where *N*_*Ind*_ = No. of individuals across all trajectories in a single inference run, and *P*_*k*_ = Probability of observing individual *k* across all trajectories in a single inference run. This is a measure of the diversity of individuals identified in an inference run.
2. Maximum probability of observing any of the two source individuals in the trajectory: $P_{\mathrm{Max}}^{Source}=\underset{k\in Source}{\max}P_{k}$, where *P*_*k*_ is defined in the preceding. This is a measure of how identifiable (either of) the source individuals are.
3. Per-SNP entropy of identified individuals, if either of the source individuals is found at that SNP: $S_{Per-SNP}(l)=$ $-\sum_{k=1}^{N_{Ind}(l)}P_{k}(l)\cdot \log P_{k}(l)$ if the source individual is found at SNP *l*, where *N*_*Ind*_(*l*) = No. of individuals at SNP *l*, and *P*_*k*_(*l*) = Probability of observing individual *k* across all trajectories at SNP *l*. This is a measure of the observed diversity in inferred individuals at each SNP position.

### Simulation of samples

We use simulated data to test the performance of PLIGHT. For the analysis of individuals known to be in the reference database, we select a single individual at random from the database. For the analysis of mosaic genotypes, we select two individuals at random. For all the individuals in a given simulation we create a genotype sample as follows. To spread out the selected SNPs across the genome, we only choose SNPs ordered along the chromosome with a probability p∼Bernoulli(1,0.003). For noise simulations, each SNP thus chosen is randomly mutated with a probability of the mutation rate per haplotype, λ, at each site (for definitions, see Supplemental Methods). If the resulting mutated genotype is heterozygous or homozygous in the alternate allele, we include the SNP. Note that this selection process is meant to mimic the case in which only alternative alleles are obtained, and reference alleles are left out. However, this can also lead to an apparent inflation of the mutation rate, as unmutated, homozygous reference alleles are left out. In our results, this inflation of the mutation rate is implicitly assumed.

For the individual-in-the-reference case, this sampling method directly provides the input data set. For the mosaic case, we divide each chromosome into two segments and assign genotypes of the two individuals, mutated in the above fashion, to the two halves.

### Mosaic genome reconstruction

We reconstruct the mosaic genomes based on the best-fit reference haplotype pairs. To do so, we grab segments from the reference VCF file corresponding to the reference haplotypes inferred in each trajectory: for $T=\;{\left\{\;j(l),k(l)\right\}}_{l=1}^{L}$, we have *L* − 1 segments to reconstruct; for each segment (defined in the following), we extract all the SNP haplotypes for reference haplotypes *j* and *k*, combining their values at each position to get the inferred genotype. The genomic segments of identified individuals at each SNP are constructed according to Start of segment $i=\frac{SNP_{i-1}+SNP_{i}}{2};$ End of segment $i=\frac{SNP_{i}+SNP_{i+1}}{2}$; where *SNP*_*i*_ = *Genomic position of SNP i*, the first segment starts at *SNP*_1_ and the last segment ends at *SNP*_*L*_. We store the mosaic reconstruction in a VCF file.

### Collection and analysis of real-world samples

#### Sample preparation

Saliva was obtained from two consenting adults, A and B, by forcible expulsion into sterile polycarbonate tubes. Saliva from Individual A and Individual B was combined, 9:1, and mixed by gentle repipetting. This combined material (300 µL) was manually applied across the entirety of the surface of a UV-irradiated (in house) 3” × 1” glass slide. This was then allowed to air dry in a PCR hood (AirClean Systems, Creedmoor, NC) for ∼18 h at room temperature.

### DNA extraction protocol

DNA extraction and purification was performed using the QIAamp DNA Investigator Kit (Qiagen 56504). Briefly, the sample was collected from the surface of the slide using a swab (4N6FLOQSwabs, Thermo Fisher Scientific 4479439) dipped first in 1 µL DNase/RNase-free water (Invitrogen 10977-015). Manufacturer protocol for total DNA extraction from surface swabs was followed. Final yields (∼460 ng) were estimated using a Qubit 4 Fluorometer and the Qubit dsDNA HS Assay Kit (Thermo Fisher Scientific Q33231).

### Whole-genome amplification and sequencing

To mimic a real-world, surreptitious gathering of samples, aliquots of extracted DNA (∼30 ng) were amplified using a multistrand displacement amplification kit (REPLI-g Advanced Single Cell Kit [Qiagen 150363]) and following, without modification, the included protocol for purified whole genomic DNA amplification. Sequencing was performed in-house at the Yale Center for Genome Analysis using an Illumina NovaSeq (HiSeq paired-end, 150bp) with total data collection corresponding to 10× coverage of the human genome.

### Genotype processing

The FASTQ files were processed through the GATK (Van der Auwera et al. 2013) pipeline for short variant discovery (https://gatk.broadinstitute.org/hc/en-us/articles/4407897446939‐‐How-to-Run-germline-single-sample-short-variant-discovery-in-DRAGEN-mode) using DRAGMAP, Illumina's open-source DRAGEN mapper (https://github.com/Illumina/DRAGMAP). In brief: DRAGMAP is used to map the reads to the hg19 reference genome; a short tandem repeat table file is composed and calibrated using the *ComposeSTRTableFile* and *CalibrateDragstrModel* programs in the GATK package; Picard tools’ (http://broadinstitute.github.io/picard/) *MarkDuplicates* algorithm is used to mark and remove read duplicates from the mapped BAM files; the GATK *HaplotypeCaller* program is used in “DRAGEN mode” to call variants; and the program *VariantFiltration* is used to carry out a hard quality-based filter using “QUAL < 10.4139”, as recommended. All variants that did not pass the hard filtering step are removed. The output is a VCF file used for the remaining analyses.

The blood genotypes were processed earlier (Gürsoy et al. 2020) according to the following protocol: the FASTQ files were mapped to the hg19 reference genome (b37 assembly) using BWA (Li and Durbin 2009), with the BAM outputs being de-duplicated using Picard tools. Finally, the deduplicated BAM files were processed utilizing GATK (DePristo et al. 2011; Van der Auwera et al. 2013) to produce variant call sets in VCF format.

The environmental-sample-based genotypes were unphased because of the noisiness and intermixture of two individuals. However, we phased the gold-standard blood tissue genotypes using the TOPMed Imputation Server (Fuchsberger et al. 2015; Das et al. 2016; Taliun et al. 2021) (details in Supplemental Methods). The environmental-sample-based genotypes serve as the noisy, sparse data set, whereas the blood-tissue-based results provide the higher-quality baseline for comparison. The VCF coordinates are defined according to human genome reference assembly GRCh37, using the reference FASTA file *human_g1k_v37.fasta.gz* (ftp.1000genomes.ebi.ac.uk/vol1/ftp/technical/reference*)*, the same as for the 1000 Genomes Phase 3 database. This registers the VCF files from the saliva samples and from 1000 Genomes in the same coordinate system, an essential requirement for *PLIGHT*.

### Mosaic genome correspondence and polygenic risk scores

We quantify the risk associated with mosaic genomes by studying the resulting imputation of query genotypes, as well as by calculating PRSs for several phenotypes in the GWAS catalog version 1.0.2 (Buniello et al. 2019). The imputation is assessed by calculating the fraction of correct SNPs and the correspondence scores *C*. The fractions, *C* scores and PRS calculation are detailed in the Supplemental Methods.

For the “Height” phenotype PRS analysis, we engineer query sets for Chromosomes 3 and 6 by identifying the “Height” GWAS SNPs on the respective chromosomes as represented in the GWAS catalog. We find all SNPs within ±2 kb of the GWAS loci, and then randomly select 90 SNPs per chromosome as our query sets. We run *PLIGHT_Iterative* with *n*_*iter*_ = 20 and *S*_*sg*_ = 300 using the full 1000 Genomes cohort, construct the mosaic genomes from the best-fit trajectories, and calculate the PRSs for those GWAS SNPs that are within the same ±2 kb windows of our query SNPs. We run the two-sided one- or two-sample *t*-tests using the base R function *t.test* (R Core Team 2021).

### Software availability

We have made our software available for download at GitHub (https://github.com/gersteinlab/PLIGHT). We provide information on the software requirements, parameter options, and examples on how to run the code. We also provide timing benchmarks for each of the algorithms. In addition, the source code is provided as a compressed folder containing the Python scripts, uploaded to the Supplemental Materials as Supplemental Code.

## Data access

All raw sequencing data generated in this study have been submitted to the NCBI Sequence Read Archive (SRA; https://www.ncbi.nlm.nih.gov/sra/) under accession number SRP466183, and to the NCBI BioProject database (https://www.ncbi.nlm.nih.gov/bioproject/) under accession number PRJNA915778. The processed VCF data generated in this study have been submitted to the NCBI database of Genotypes and Phenotypes (dbGaP; https://www.ncbi.nlm.nih.gov/gap/) under accession number phs003166.v1.p1.

## Supplementary Material

Supplement 1

Supplement 2

Supplement 3
