# Supplementary material for: Assessing and mitigating privacy risks of sparse, noisy genotypes by local alignment to haplotype databases
Source: Genome Res. 2023 Dec;33(12):2156–73. doi: 10.1101/gr.278322.123 (PMC10760520; doi:10.1101/gr.278322.123)
Supplement: Supplement 3 [file Supplemental_Material.pdf]

Supplemental Materials

Assessing and mitigating privacy risk of sparse, noisy genotypes by local alignment to haplotype databases

Prashant S. Emani[1,2], Maya N. Geradi[1,2], Gamze Gürsoy[1,2,3,4], Monica R. Grasty[2], Andrew Miranker[2], Mark B. Gerstein[1,2,5,6*]

[1]Program in Computational Biology and Bioinformatics, Yale University, New Haven, CT 06520, USA
[2]Department of Molecular Biophysics and Biochemistry, Yale University, New Haven, CT 06520, USA
[3]Current Address: Department of Biomedical Informatics, Columbia University, New York, NY 10032, USA
[4]Current Address: New York Genome Center, New York, NY, 10013, USA
[5] Department of Computer Science, Yale University, New Haven, CT 06520, USA
[6] Department of Statistics & Data Science, Yale University, New Haven, CT 06520, USA.

*Corresponding author: pi@gersteinlab.org

Table of Contents

Supplemental Figures *4*

Supplemental Figure S1. 4

Supplemental Figure S2. 5

Supplemental Figure S3. 6

Supplemental Figure S4. 7

Supplemental Figure S5. 9

Supplemental Figure S6. 11

Supplemental Figure S7. 13

Supplemental Figure S8. 15

Supplemental Figure S9. 16

Supplemental Figure S10. 17

Supplemental Figure S11. 16

Supplemental Figure S12. 16

Supplemental Figure S13. 20

Supplemental Figure S14. 21

Supplemental Files *22*

Supplemental File S1 22

Supplemental Tables *23*

Supplemental Table S1 23

Supplemental Table S2 24

Supplemental Table S3 25

Supplemental Methods *26*

Simulation of the degree of correlation of randomly selected SNPs. 26

Li-Stephens model and associated biological parameters 26

Hidden Markov Model optimization 28

Matrix methods used in the modified Viterbi algorithm. 30

Truncation scheme. 31

Imputation of blood tissue sample genotypes 32

Mosaic genome correspondence and polygenic risk scores 32

Correspondence score. 32

Polygenic risk score calculation. 32

Supplemental Results *34*

Simulation of the degree of correlation of randomly selected SNPs. 34

Supplement to Section "Identification of individuals known to be within a database". **34**
Supplement to Section "Identification of individuals from contaminated samples". **35**
Supplement to Section "Truncated algorithm". **35**
Supplement to "Predicting genotypes at GWAS loci and polygenic risk score (PRS) analysis" **36**
Supplement to "Sanitization of SNPs based on Inferred Trajectories" **37**
References ***38***

Supplemental Figures

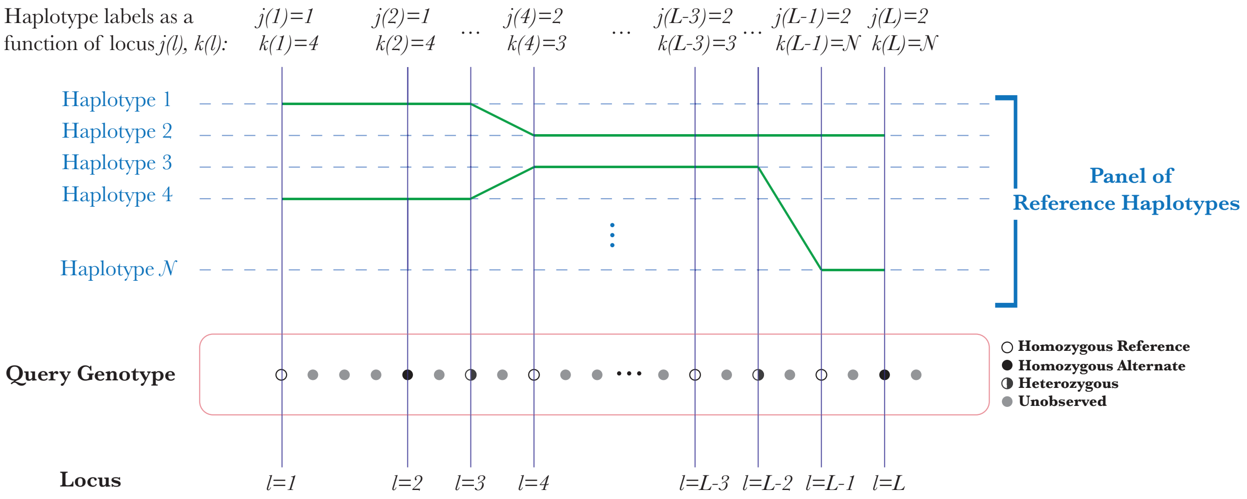

Supplemental Figure S1. Explanation of the j(l) indexing as a function of the query locus l, for one trajectory. The best-fit states in the HMM consist of a pair of haplotype labels at each locus loci $l = \{1,2,\cdots,L\}$: these are the names of the reference haplotypes that best match the query SNPs over certain genomic tracts, and the trajectory is the sequence of all such pairs of haplotype labels, $\mathcal{T} = \{j(l),k(l)\}_{l=1}^{L}$.

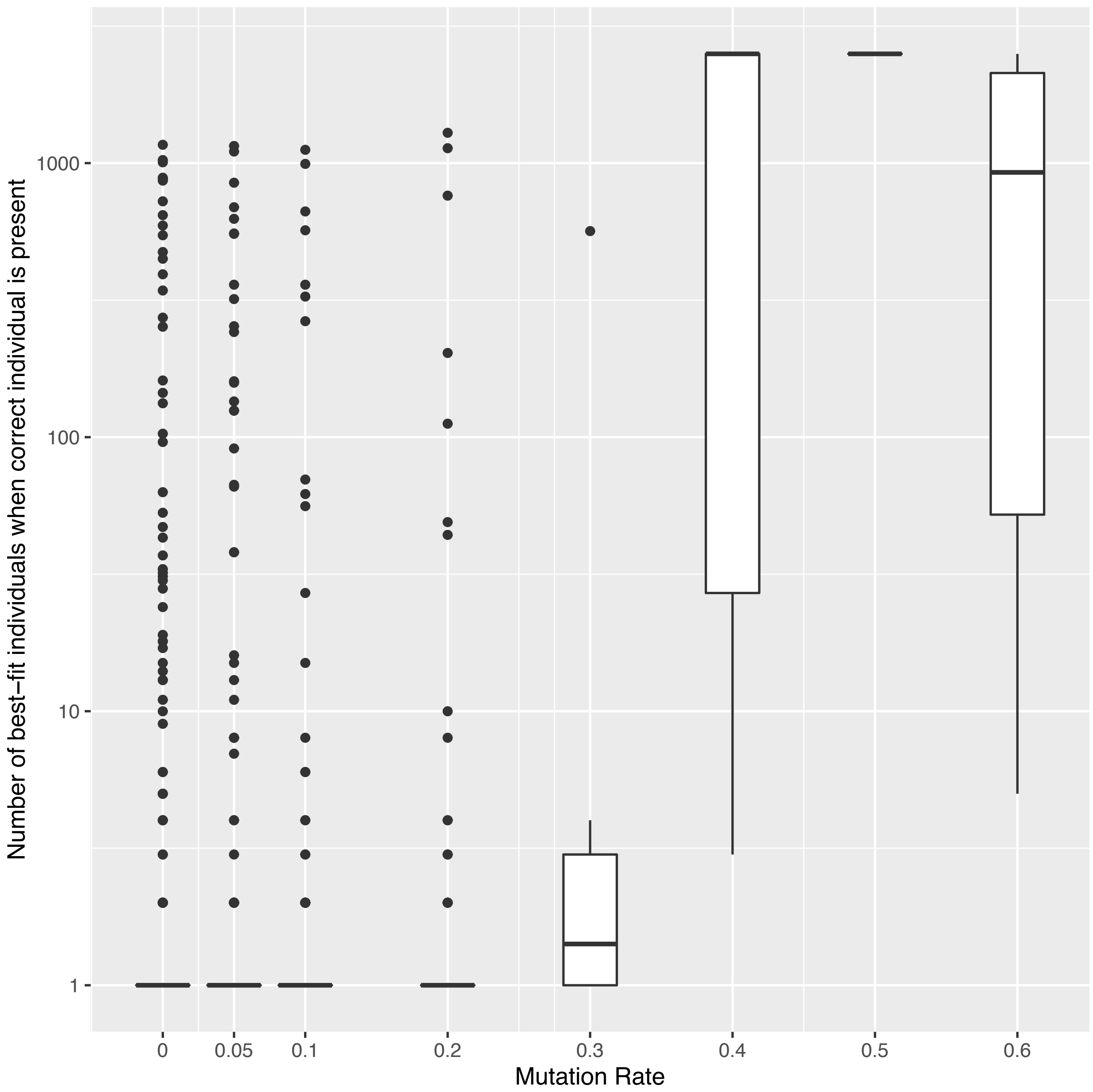

Supplemental Figure S2. Individual-in-database study: Plot of the distribution of the number of individuals in the best-fit trajectory set for the replicate simulations where the query individual is present in the reference database, as a function of the mutation rate (see **Supplemental Results** for details). Note that only the bottom of the boxplot for the mutation rate = 0.3 case reaches 1 (which is the unique identification scenario) whereas the higher mutation rates have no instances where the correct individual is uniquely found.

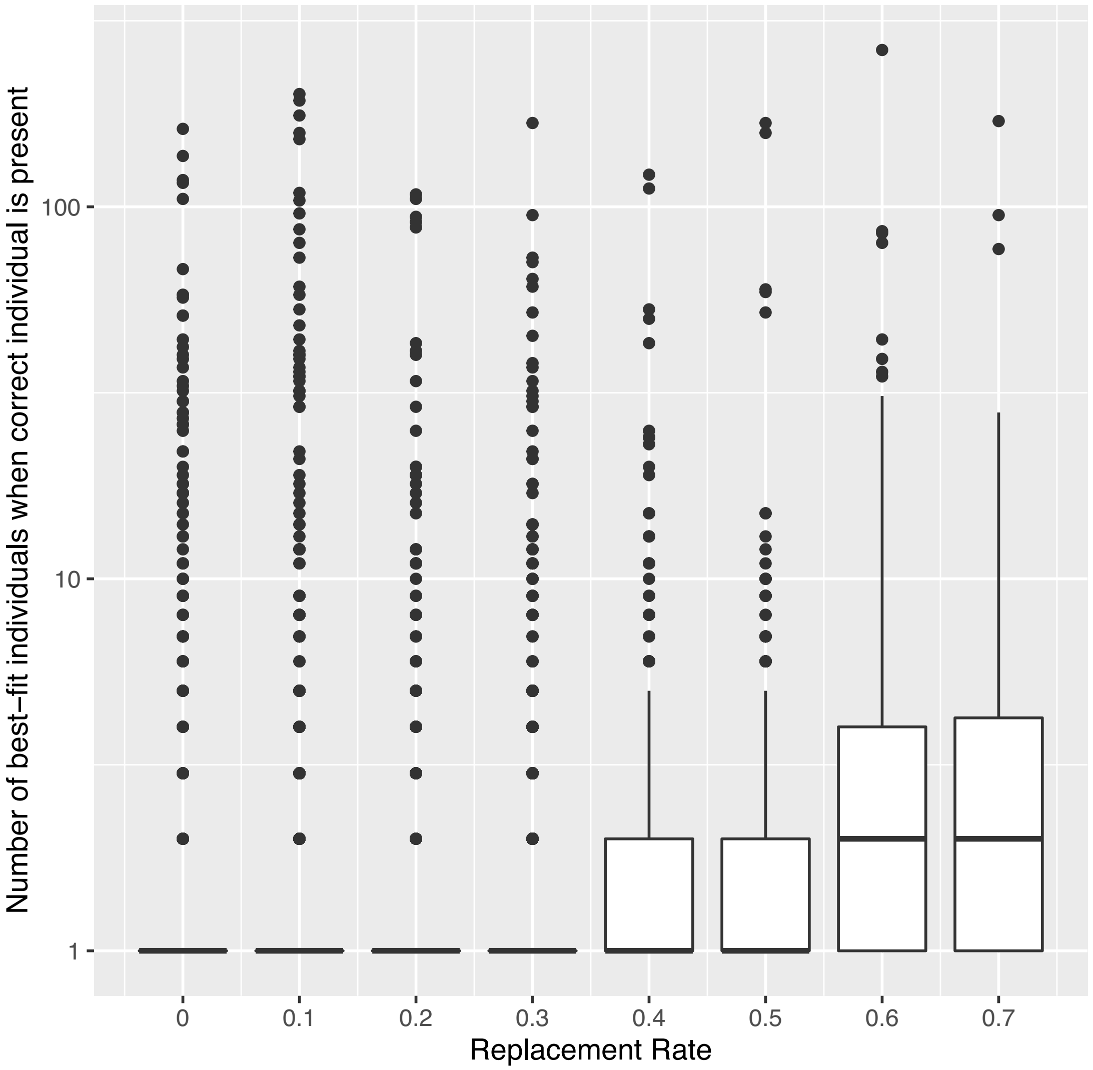

Supplemental Figure S3. Contamination study: Plot of the distribution of the number of individuals in the best-fit trajectory set for the replicate simulations (error rate = 0.0) where the query individual is present in the reference database, as a function of the replacement rate in the contamination study (see **Supplemental Results** for details). Note that a unique and correct identification corresponds to the overlap of the bars with a y-axis value of 1.

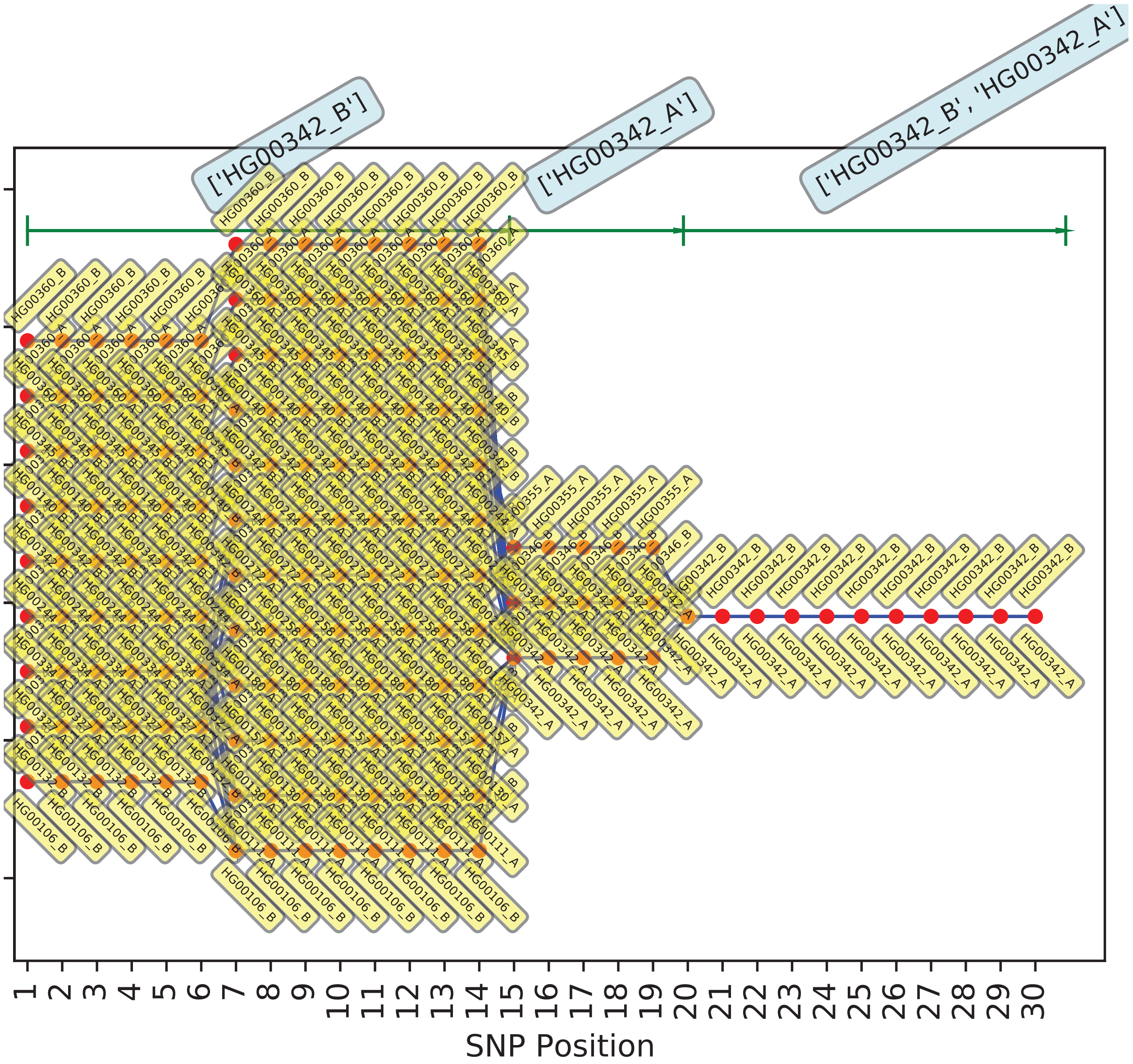

Supplemental Figure S4. Best-fit genotypic trajectories from *PLIGHT_Exact* for the diploid mosaic genome of HG00360+HG00342 constructed across 30 SNPs each for Chromosome 21 (corresponding results for Chromosomes 1 and 2 in Figures 3A-B). The composition of the best-fit pair of haplotypes at each locus is depicted by two yellow tags, one below and one above the red dots.

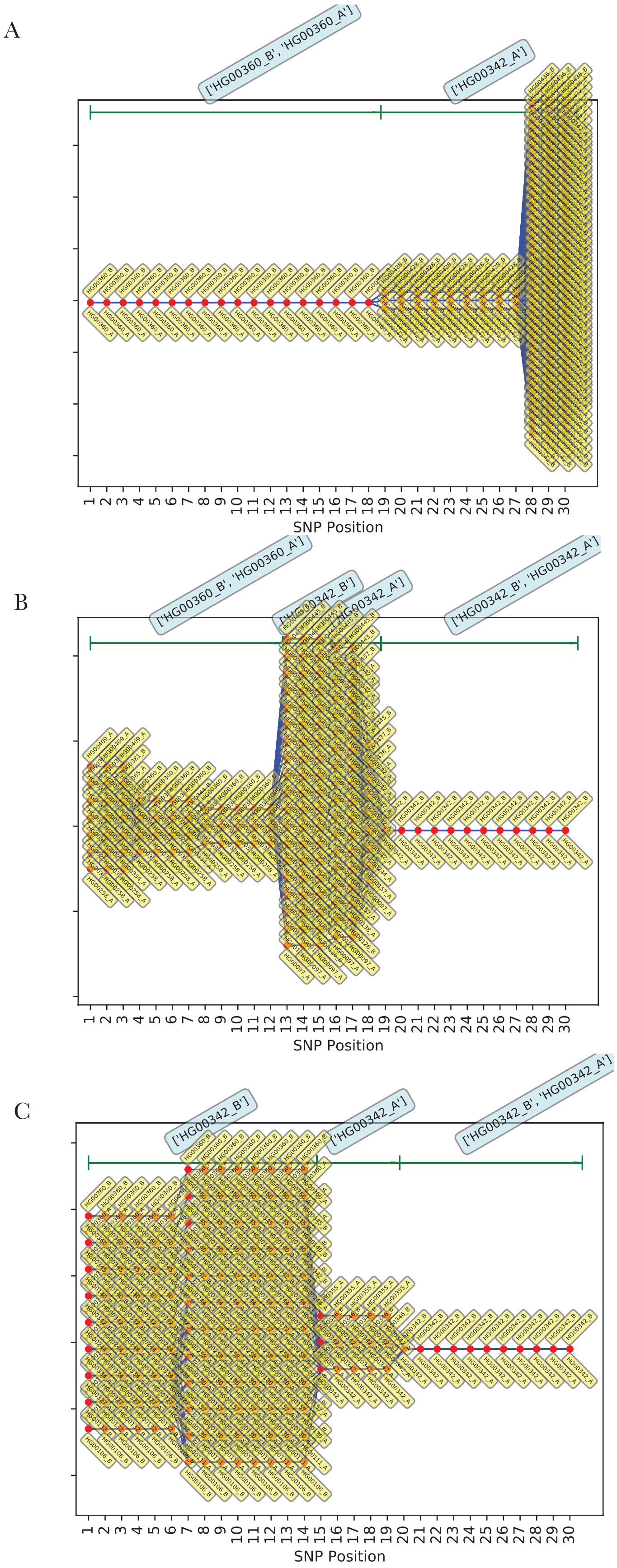
A
['HG00360_B', 'HG00360_A']
['HG00342_A']
SNP Position
B
['HG00360_B', 'HG00360_A']
['HG00342_B']
['HG00342_A']
['HG00342_B', 'HG00342_A']
SNP Position
C
['HG00342_B']
['HG00342_A']
['HG00342_B', 'HG00342_A']
SNP Position

Supplemental Figure S5. Best-fit genotypic trajectories from *PLIGHT_Exact* for the diploid mosaic genome of HG00360+HG00342 constructed across 30 SNPs each for chromosome with recombination rate = 1.0 cM/Mb: (A) Chromosome 1; (B) Chromosome 2; (C) Chromosome 21. The composition of the best-fit pair of haplotypes at each locus is depicted by two yellow tags, one below and one above the red dots.

A Truncation factor = 0.005

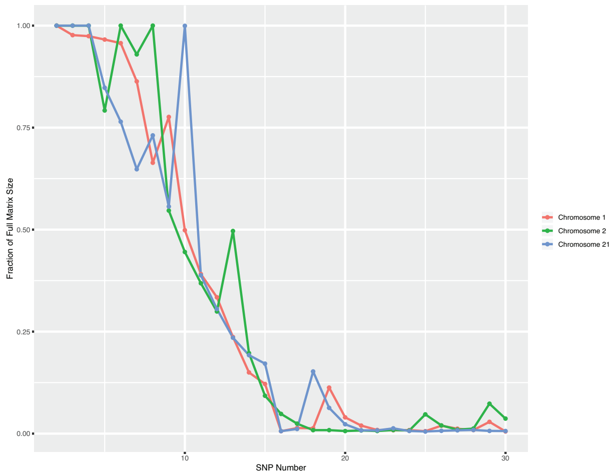

B Truncation factor = 0.02

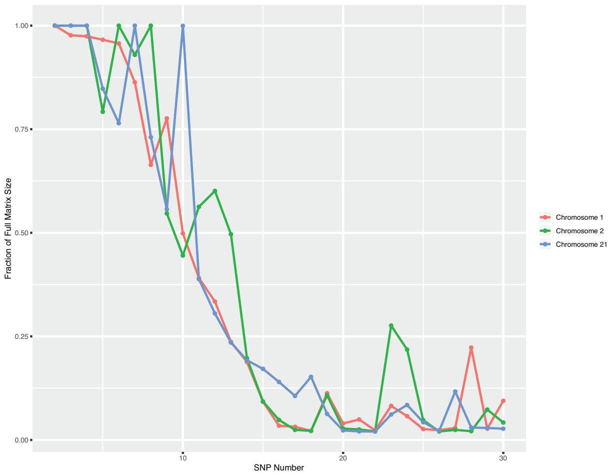

C Chromsome 2, Truncation factor = 0.005

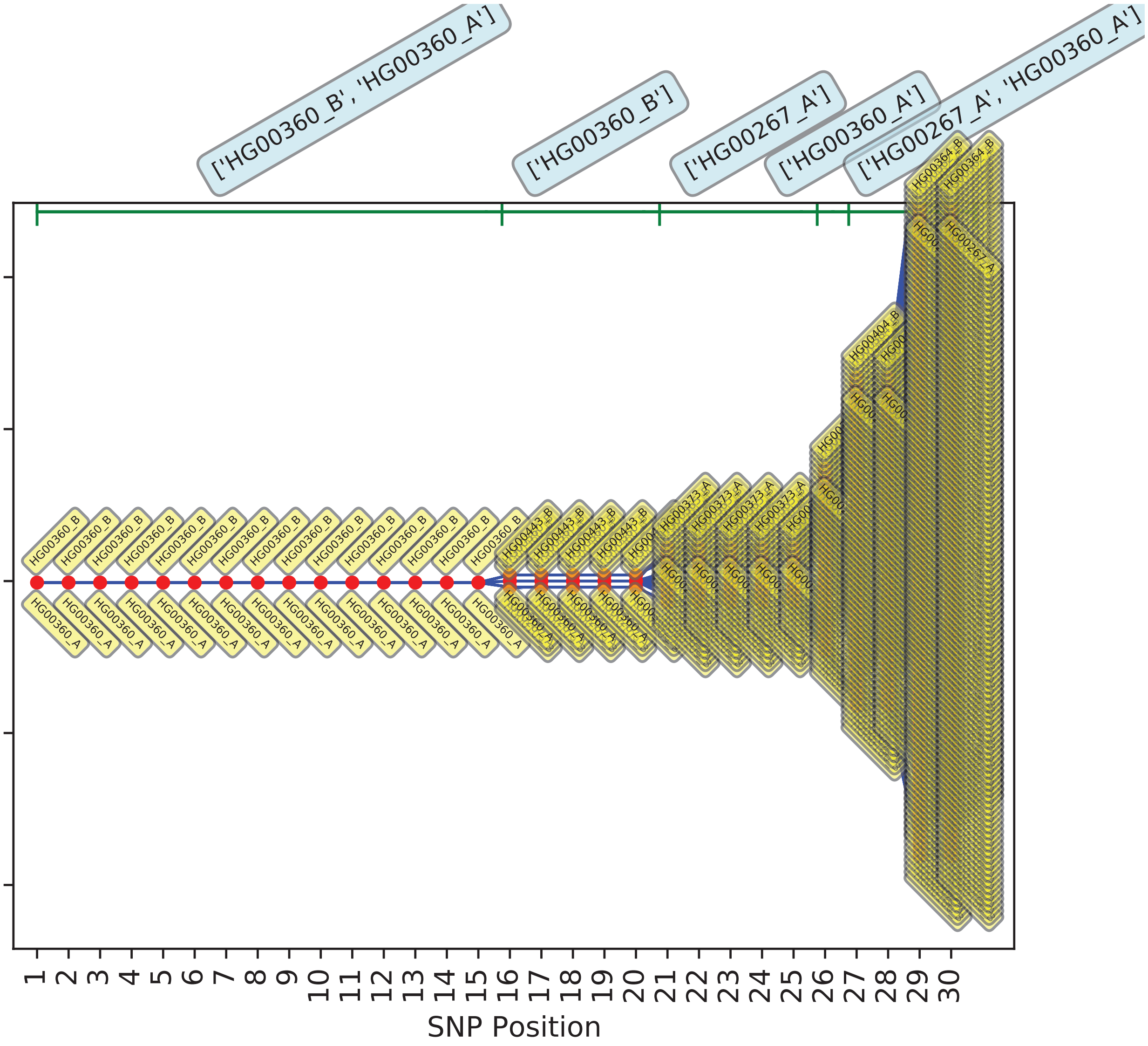

Supplemental Figure S6. Results for the *PLIGHT_Truncated* algorithm applied to the same query SNP set as for **Figure 3**. (A) Fraction of the full matrix size at each step (i.e. SNP) in the HMM sequence, for a final truncation factor of $f = 0.005$. (B) Fraction of the full matrix size at each step (i.e. SNP) in the HMM sequence, for a final truncation factor of $f = 0.02$. (C) Trajectory for Chromosome 2, shown to illustrate a case where truncation results in different trajectories relative to the exact case (compare to **Figure 3B**). The composition of the best-fit pair of haplotypes at each locus is depicted by two yellow tags, one below and one above the red dots.

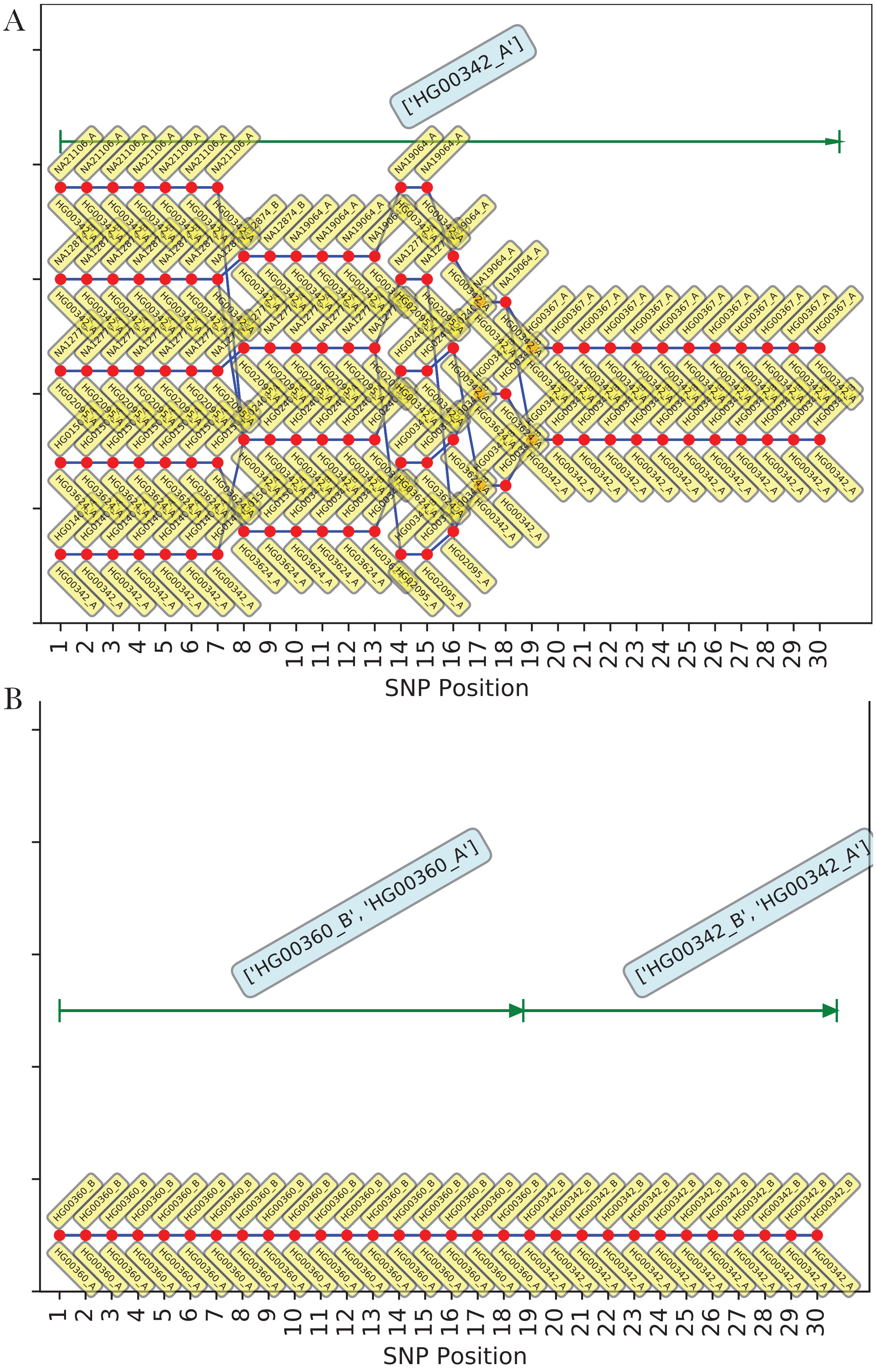
A
['HG00342_A']
1 2 3 4 5 6 7 8 9 10 11 12 13 14 15 16 17 18 19 20 21 22 23 24 25 26 27 28 29 30
SNP Position
B
['HG00360_B', 'HG00360_A']
['HG00342_B', 'HG00342_A']
1 2 3 4 5 6 7 8 9 10 11 12 13 14 15 16 17 18 19 20 21 22 23 24 25 26 27 28 29 30
SNP Position

Supplemental Figure S7. Consensus genotypic trajectories from *PLIGHT_Iterative* for the diploid mosaic genome of HG00360+HG00342 constructed across 30 SNPs in Chromosome 1, where the consensus score is evaluated by weighting the haplotypes in each trajectory in proportion to their occurrence across all three chromosomes. The composition of the best-fit pair of haplotypes at each locus is depicted by two yellow tags, one below and one above the red dots. (A) $\mathrm{n_{iter}} = 20$, replicate 2; (B) $\mathrm{n_{iter}} = 30$, replicate 2. Shown at the top of each panel are the most frequent haplotypes within each segment indicated. The corresponding first replicates are shown in Figure 4 of the main manuscript.

A

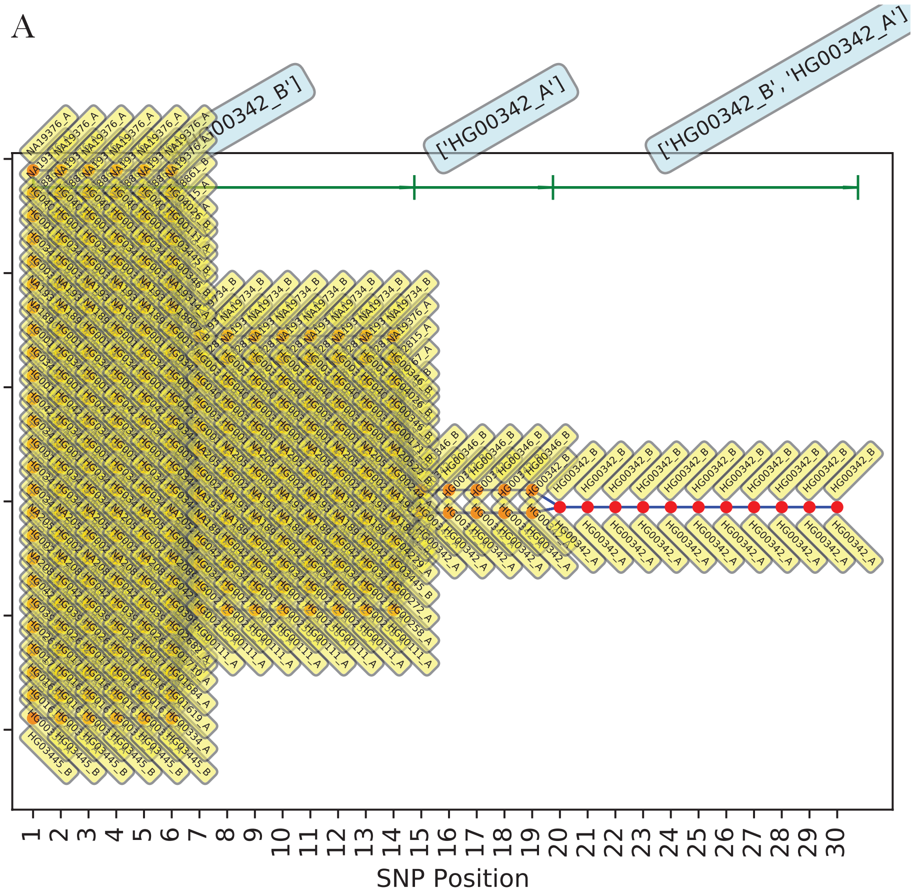

['HG00342_B']
['HG00342_A']
['HG00342_B', 'HG00342_A']
1 2 3 4 5 6 7 8 9 10 11 12 13 14 15 16 17 18 19 20 21 22 23 24 25 26 27 28 29 30
SNP Position

B

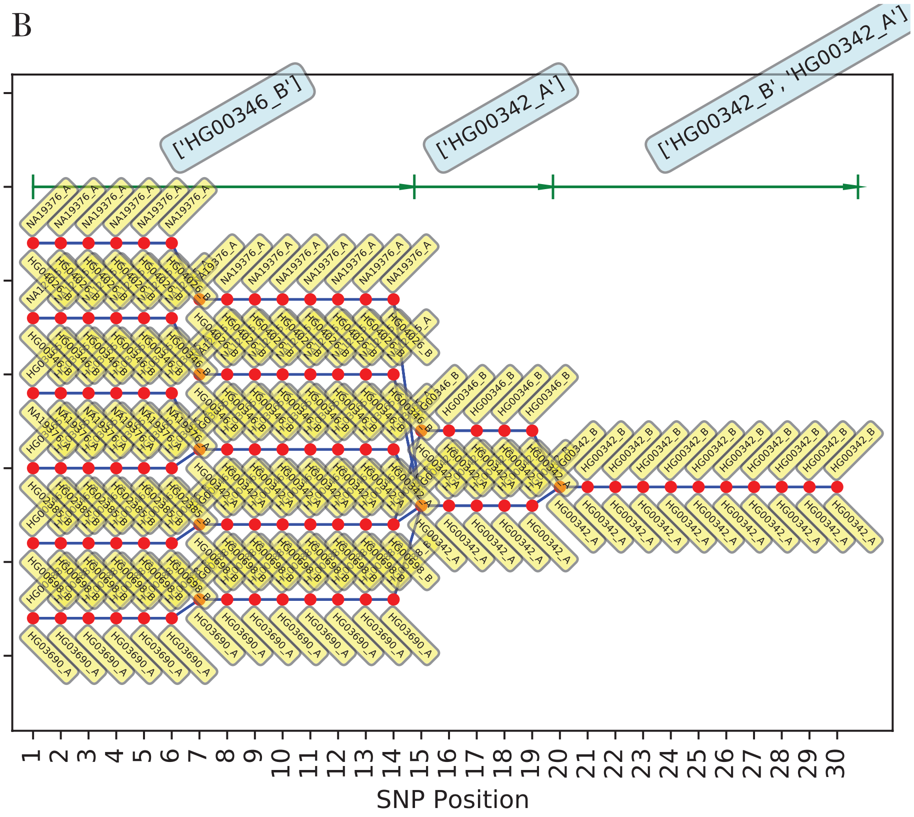

['HG00346_B']
['HG00342_A']
['HG00342_B', 'HG00342_A']
1 2 3 4 5 6 7 8 9 10 11 12 13 14 15 16 17 18 19 20 21 22 23 24 25 26 27 28 29 30
SNP Position

Supplemental Figure S8. Consensus genotypic trajectories from *PLIGHT_Iterative* for the diploid mosaic genome of HG00360+HG00342 constructed across 30 SNPs in Chromosome 21, where the consensus score is evaluated by weighting the haplotypes in each trajectory in proportion to their occurrence across all three chromosomes. The composition of the best-fit pair of haplotypes at each locus is depicted by two yellow tags, one below and one above the red dots. (A) $\mathrm{n}_{\mathrm{iter}} = 20$, replicate 1; (B) $\mathrm{n}_{\mathrm{iter}} = 30$, replicate 1. Shown at the top of each panel are the most frequent haplotypes within each segment indicated. The corresponding first replicates are shown in Figure 4 of the main manuscript. Panel A is an example of a successful identification of the two component individuals, while Panel B shows a case where only one of the two individuals is found.

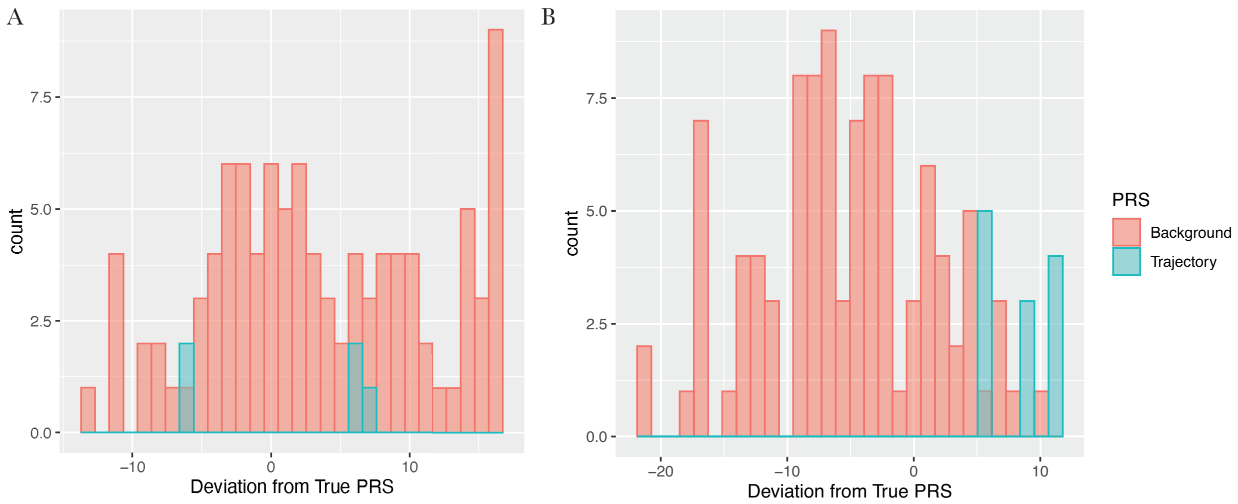

Supplemental Figure S9. Histograms of the deviation values from the true PRS scores for the Height GWAS analysis, shown for both inferred trajectories and background individuals. (A) Results for Chromosome 3 (red = Background PRS - True PRS, blue = Trajectory PRS - True PRS); (B) Results for Chromosome 6 (red = Background PRS - True PRS, blue dashed line = Trajectory PRS - True PRS).

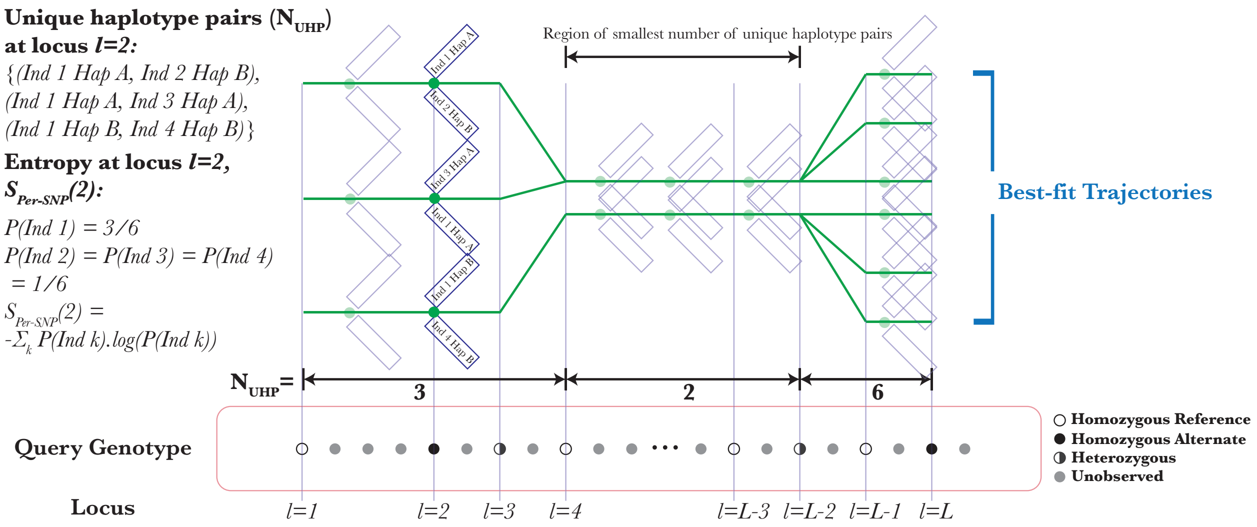

Supplemental Figure S10. Schematic of the metrics used in the sanitization scheme. In green is shown an example of a set of best-fit parallel trajectories. At locus $l = 2$, the figure shows examples of the identified haplotype pairs for this particular set of trajectories. On the left, the figure includes an explanation of how the unique haplotype pairs are counted, as wells as how the entropy across individuals per SNP is calculated at a particular locus. The figure also includes a marking of the region of the smallest number of unique haplotype pairs, which is then used in the sanitization procedure outlined in the paper.

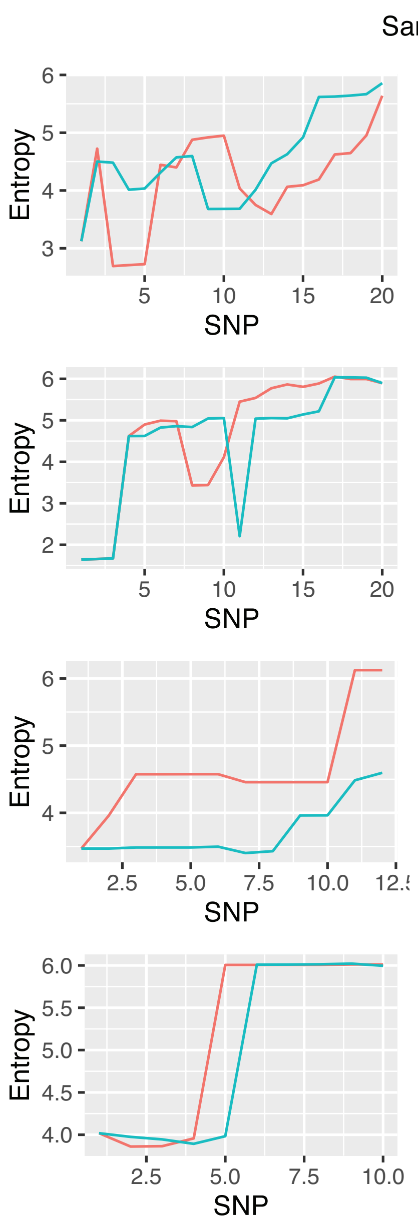

Supplemental Figure S11. Plot of the total entropy of individuals across all trajectories (S_{Ind} defined in the main manuscript) for each of 10 independent query SNP sets, for the two different sanitization strategies. The entropy is plotted as a function of the number of SNPs removed, which varies from run to run.

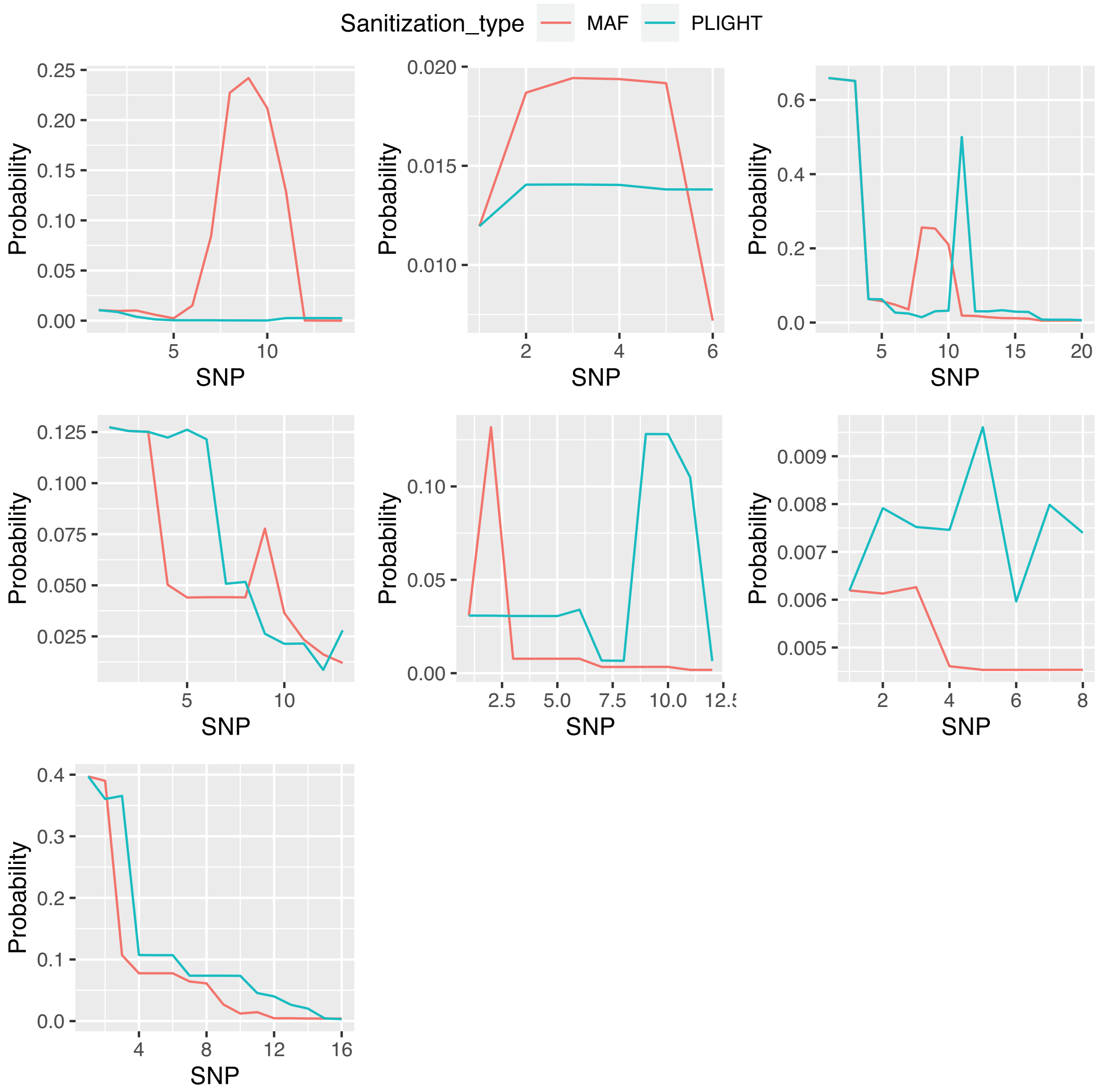

Supplemental Figure S12. Plot of the maximum probability of finding any of the source individuals in all trajectories (P_{Max}^{Source} defined in the main manuscript) for each of 7 independent query SNP sets, for the two different sanitization strategies. 10 sets were run originally but 3 of the runs did not find the underlying source individuals. The probability is plotted as a function of the number of SNPs removed, which varies from run to run.

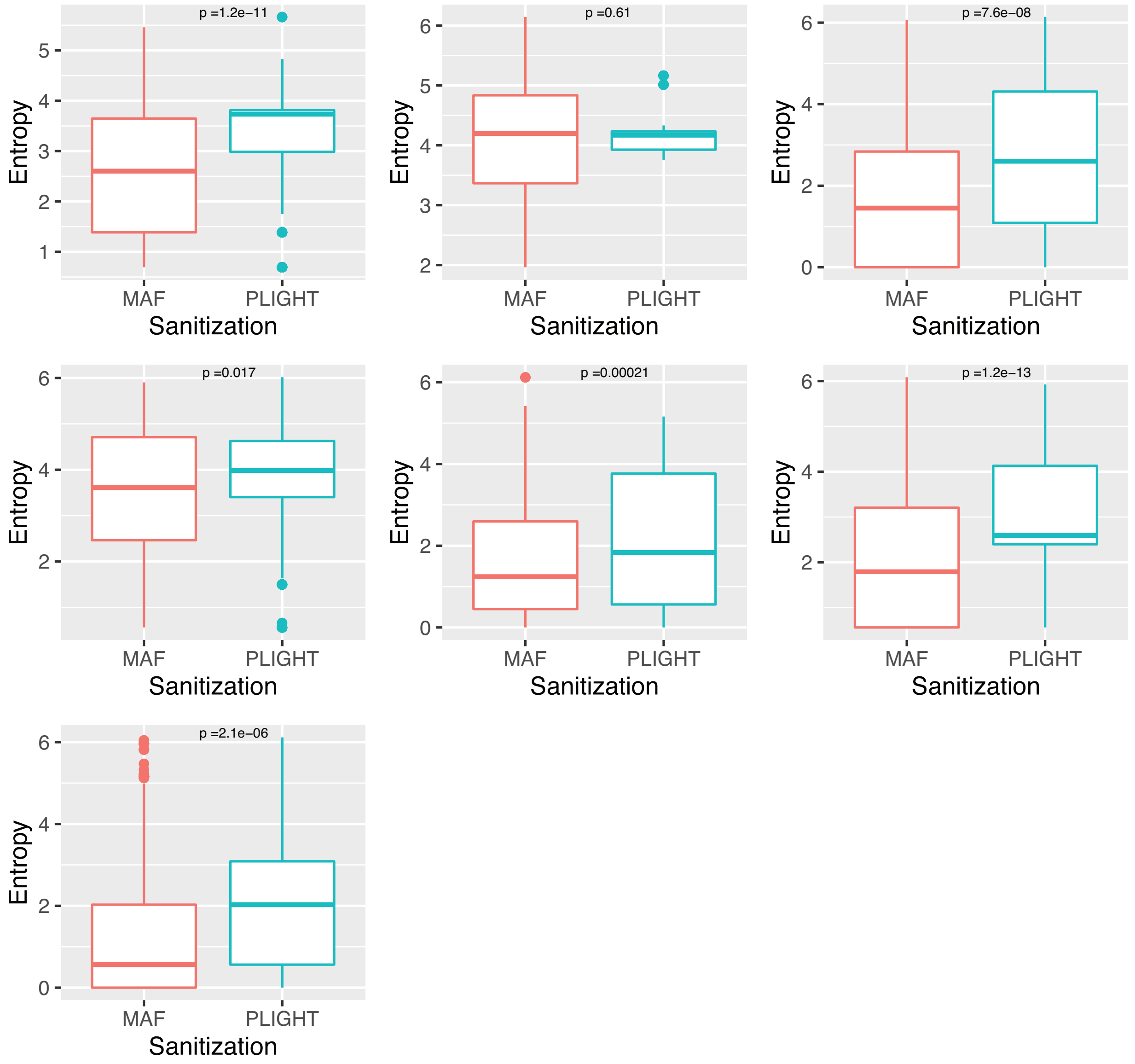

Supplemental Figure S13. Plot of the per-SNP entropy for those SNPs containing any of the source individuals in all trajectories ($S_{Per-SNP}(i)$ defined in the main manuscript) for each of 7 independent query SNP sets, as a function of the two different sanitization strategies. 10 sets were run originally but 3 of the runs did not find the underlying source individuals. All the per-SNP entropies are grouped together according to the sanitization strategy employed in this figure (see Supplemental Figure S12 for all the removed SNPs treated separately). P-values are shown for the comparison of means between the two distributions based on the Wilcoxon two-sample test.

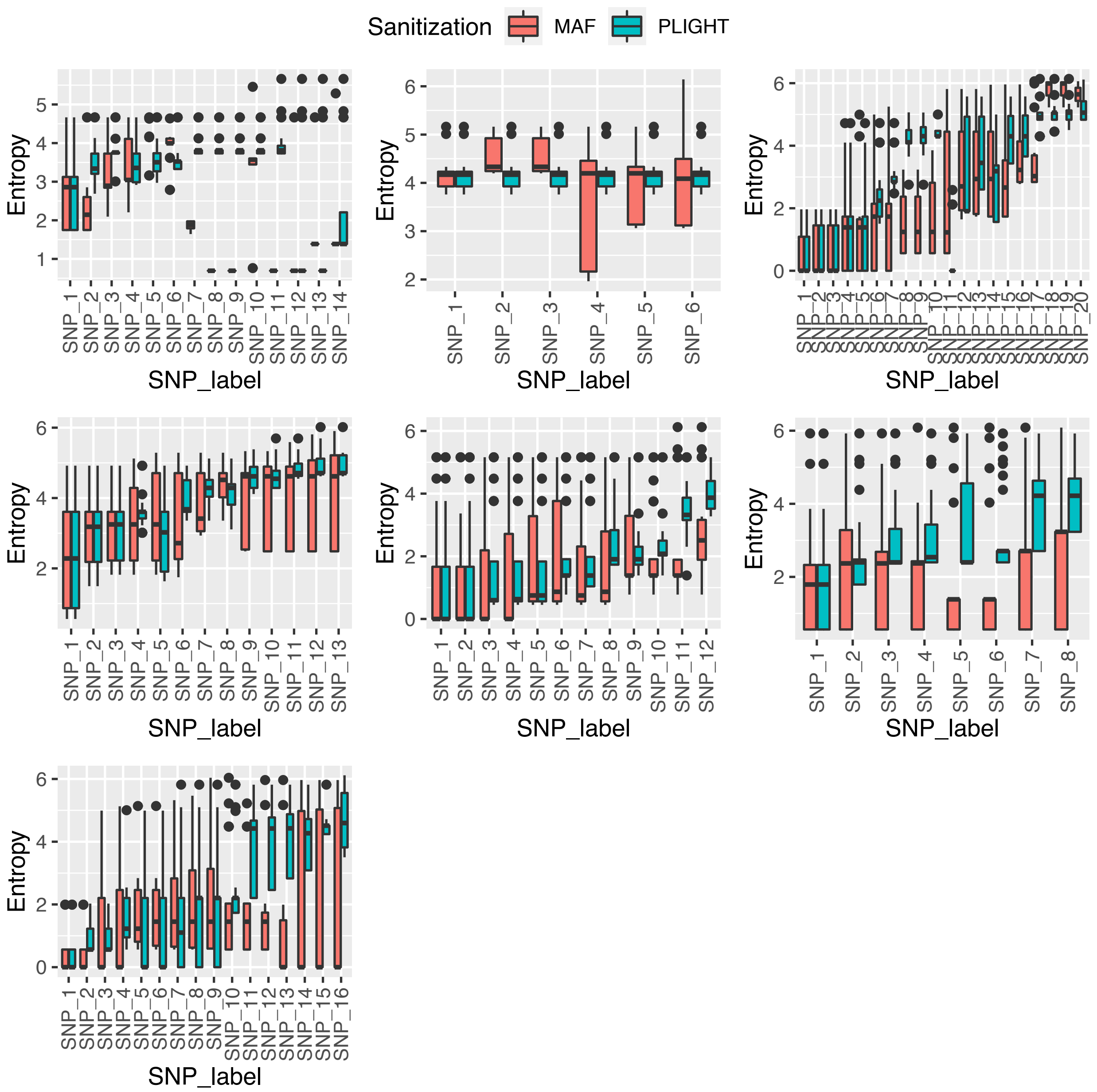

Supplemental Figure S14. Plot of the per-SNP entropy for those SNPs containing any of the source individuals in all trajectories ($S_{Per-SNP}(i)$ defined in the main manuscript) for each of 7 independent query SNP sets, as a function of the individual SNPs removed. 10 sets were run originally but 3 of the runs did not find the underlying source individuals.

Supplemental Files

Supplemental File S1. This file reports the results of the contamination analysis, for the different mutation rates reported in Table 2 of the main manuscript and for the two populations used for the contamination simulation: (a) a "General" population where contamination was by samples randomly selected from across the 1000 Genomes cohort and (b) a "CDX" population where contamination was by samples randomly selected from the CDX population specifically. For each mutation rate, we report: (1) The minimum number of SNPs required for correct identification, in all runs (out of a maximum of 30) where the correct source individual was found; (2) The difference in the log probability of the observed SNPs between the HMM model and an independent SNP model with genotype frequencies ($\frac{log(P_{HMM})-log(P_{GF})}{N_{SNPs}}$ defined in the main text), in all runs (out of a maximum of 30) where the correct source individual was found; (3) The average minimum number of SNPs and the standard deviation (using the numbers quoted above); and (4) The average difference in the log probability and the standard deviation (using the numbers quoted above).

Supplemental Tables

Supplemental Table S1. Table of conditional probabilities for the observed genotypes $G_{q,l}$ based on the sum of two reference haplotypes, $Z^{(1)}_{j(l),l} + Z^{(2)}_{k(l),l}$, as a function of the mutation rate per haplotype λ.

		$G_{q,l}$		
		**0**	**1**	**2**
$Z^{(1)}_{j(l),l} + Z^{(2)}_{k(l),l}$	**0**	$(1-\lambda)^2$	$2\lambda(1-\lambda)$	λ^2
	**1**	$\lambda(1-\lambda)$	$\lambda^2 + (1-\lambda)^2$	$\lambda(1-\lambda)$
	**2**	λ^2	$2\lambda(1-\lambda)$	$(1-\lambda)^2$

Supplemental Table S2. PRS matching scores for the true query genome to the best-fit mosaic trajectories for the simulated mosaic HG00360 + HG00342 SNPs across the regions of chromosomes ranging from the first observed SNP to the last observed SNP. Corresponding matching scores for the true sample to the background genomes are shown in parentheses. Four different cosine similarity scores are chosen with the aim of elucidating potentially subtle differences in the matching depending on the choice of phenotypes or method of averaging: (1) **ALL** = Cosine similarity of the true sample score relative to the mean score, for all traits; (2) **> 1 SNP** = Cosine similarity of the true sample score relative to the mean score, for all non-zero traits with more than one GWAS SNP; (3) **PRS > 2** = Cosine similarity of the true sample score relative to the mean score, for all non-zero traits where the absolute value of the *Z*-score of the true PRS > 2; (4) **Compare, then average** = Cosine similarity of the true sample score **relative to the score of each trajectory, subsequently averaged**, for all traits. Non-zero traits are those for which the true sample has a non-zero PRS.

Similarity score	Chromosome 1	Chromosome 2	Chromosome 21
ALL: *Best-fit mosaics (background individuals)*	0.9983 (0.9982) for 343 non-zero traits	0.9694 (0.8575) for 225 non-zero traits	0.9213 (0.9478) for 131 non-zero traits
> 1 SNP: *Best-fit mosaics (background individuals)*	0.9288 (0.7637) for 197 traits	0.9111 (0.7824) for 93 traits	0.9517 (0.9526) for 53 traits
PRS > 2: *Best-fit mosaics (the background individuals were used to calculate the Z-scores)*	0.8143 for 12 traits	0.7683 for 14 traits	0.9712 for 17 traits
Compare, then average: *Best-fit mosaics (background individuals)*	0.9978 (0.7113)	0.9276 (0.7811)	0.8961 (0.8402)

Supplemental Table S3. PRS matching scores for the true query genome to the best-fit mosaic trajectories for 30 and 90 environmental sample SNPs across the regions of chromosomes ranging from the first observed SNP to the last observed SNP. Corresponding matching scores for the true sample to the background genomes are shown in parentheses. Four different cosine similarity scores are chosen with the aim of elucidating potentially subtle differences in the matching depending on the choice of phenotypes or method of averaging: (1) **ALL** = Cosine similarity of the true sample score relative to the mean score, for all traits; (2) **> 1 SNP** = Cosine similarity of the true sample score relative to the mean score, for all non-zero traits with more than one GWAS SNP; (3) **PRS > 2** = Cosine similarity of the true sample score relative to the mean score, for all non-zero traits where the absolute value of the *Z*-score of the true PRS > 2; (4) **Compare, then average** = Cosine similarity of the true sample score **relative to the score of each trajectory, subsequently averaged**, for all traits. Non-zero traits are those for which the true sample has a non-zero PRS.

**Cosine similarity metric**	**Chromosome 3**	**Chromosome 6**
ALL: *Best-fit mosaics (background individuals)*	**30-SNP-case:** 0.82 (0.98) for 569 non-zero traits	**30-SNP-case:** 0.96 (0.96) for 668 non-zero traits
	**90-SNP-case:** 0.97 (0.97) for 587 non-zero traits	**90-SNP-case:** 0.93 (0.96) for 672 non-zero traits
> 1 SNP: *Best-fit mosaics (background individuals)*	**30-SNP-case:** -0.03 (0.9996) for 314 traits	**30-SNP-case:** 0.981 (0.997) for 380 traits
	**90-SNP-case:** 0.9997 (0.9995) for 320 traits	**90-SNP-case:** 0.995 (0.997) for 384 traits
PRS > 2: *Best-fit mosaics (the background individuals were used to calculate the Z-scores)*	**30-SNP-case:** 0.61 for 52 traits	**30-SNP-case:** -0.39 for 63 traits
	**90-SNP-case:** 0.46 for 58 traits	**90-SNP-case:** -0.34 for 61 traits
Compare, then average: *Best-fit mosaics (background individuals)*	**30-SNP-case:** 0.82 (0.90)	**30-SNP-case:** 0.48 (0.73)
	**90-SNP-case:** 0.96 (0.90)	**90-SNP-case:** 0.94 (0.75)

Supplemental Methods

Simulation of the degree of correlation of randomly selected SNPs.

We have carried out an analysis into how often SNPs may be expected to be correlated even in the specific case of a random selection across a chromosome. We simply made a random selection of N SNPs across a chromosome out of all possible ones, and then used the program *LDlinkR* (Myers et al. 2020) (https://github.com/CBIIT/LDlinkR) to calculate the pairwise LD for the selected SNPs using the (default) 1000 Genomes (The 1000 Genomes Project Consortium 2015) populations. We ran 1,000 simulations of this process, calculating linkage disequilibrium (LD) under 5 superpopulations, "AFR","AMR","EAS","EUR","SAS", and identifying the number of times at least one pair of SNPs occurs for which $R^2 > 0.5$. That is, if a single pair occurs for a single superpopulation, we count it as a score of 1.

Li-Stephens model and associated biological parameters

For clarity, we summarize the primary aspects of the Li-Stephens model as applicable to the work herein. Let $G_q = \{G_{q,l}\}_{l=1}^{L}$ be the genotypes of a query individual q, observed at SNP loci $l = \{1,2,\cdots,L\}$. The probability of observing such an individual given a space of reference haplotypes $H = \{Z_{j,l}\}_{l=1;j=1}^{l=L_{Ref};j=N}$ (L_{Ref} = total number of genotyped sites in the reference genomes, N = total number of haplotypes in the reference database) is:

$$P(G_q|H) = \sum_{Z_j^{(1)},Z_k^{(2)}} P\left(G_q\middle|Z_j^{(1)},Z_k^{(2)}\right).P\left(Z_j^{(1)},Z_k^{(2)}\middle|H\right) \qquad \text{(S1)}$$

where the set of all possible haplotypes at the observed loci on the two chromosomes is given by $Z_j^{(\alpha)} = \left\{Z_{j(l),l}^{(\alpha)}\right\}_{l=1}^{L}$, with $Z_{j(l),l}^{(\alpha=1,2)}$ being the haplotype at position l, and j being the index of the sampled haplotype. We treat the haplotype index $j(l)$ as a function of l, as it is possible for the choice of reference haplotype to be different at each locus; that is, in the haplotype matching process, recombination between reference haplotypes may occur from one observed locus to the next (see **Supplemental Fig. S1**). The second subscript explicitly indicates that, for reference haplotype $j(l)$, we select the genotype at locus l.

The assumption in the current iteration of the algorithm is that the observed genotypes and the reference haplotypes are registered with respect to the same, linear reference genome. This enables a simpler matching of reference haplotypes to observed genotypes. For data structures such as personal genomes and graph genomes, additional genotype matching strategies would need to be incorporated, but the conceptual framework of searching through recombining haplotypes would be the same. In general, the set of genotyped sites does not have to perfectly overlap with the set of reference haplotype sites because of rare SNPs in an individual's genotype or differences in genotyping arrays. However, for the purposes of this study we only consider genotyped sites that overlap with those of the reference haplotypes, especially given our interest in determining the identification power of common SNPs. In case of the presence of structural variants overlapping SNP loci, we allow for missing loci in any of the reference haplotypes. We thus consider the reference haplotypes as providing the complete

search space, especially in light of the constantly growing genetic databases available for comparison. We avoid making explicit assumptions of population membership and statistics for the query individual with the belief that, beyond the implicit assumptions of the chosen reference set, this will enable more unbiased estimates of kinship and genotypic similarity.

$P\left(Z_j^{(1)}, Z_k^{(2)} \middle| H\right)$ contains information on the “trajectories” through haplotype space that emerge from the reference set: it is the probability of obtaining a given set of haplotype observations at all the query loci. In general, this is not a simple measure of the frequencies of entire reference haplotypes (unless the query genotype is known to be in the reference set) due to the possibility of recombination. Recombination is incorporated into the analysis in the expressions for the transition probabilities from one query site to the next. Using results of Li and Stephens (Li and Stephens 2003) and Marchini et al (Marchini et al. 2007), we have

$$P\left(Z_j^{(1)}, Z_k^{(2)} \middle| H\right) = P\left(Z_{j(1),1}^{(1)}, Z_{k(1),1}^{(2)} \middle| H\right) \prod_{l=1}^{L-1} P\left(\left\{Z_{j(l),l}^{(1)}, Z_{k(l),l}^{(2)}\right\} \to \left\{Z_{j(l+1),l+1}^{(1)}, Z_{k(l+1),l+1}^{(2)}\right\} \middle| H\right) \tag{S2}$$

where $P\left(Z_{j(1),1}^{(1)}, Z_{k(1),1}^{(2)} \middle| H\right)$ is the probability of observing a given set of haplotypes at the first query locus. This is often drawn from a uniform distribution across all haplotype pairs, but could be modified if prior knowledge on the membership of the query individual in a particular subpopulation is available. All terms are written with the conditional dependence on the set of reference haplotypes, H, made explicit. The transition from one site to the next is given by

$$P\left(\left\{Z_{j(l),l}^{(1)}, Z_{k(l),l}^{(2)}\right\} \to \left\{Z_{j(l+1),l+1}^{(1)}, Z_{k(l+1),l+1}^{(2)}\right\} \middle| H\right) = \begin{cases} \left(e^{-\frac{\rho_l}{N}} + \frac{1-e^{-\frac{\rho_l}{N}}}{N}\right)^2 & if\ neither\ haplotype\ changes\ from\ site\ l\ to\ l+1 \\ \left(e^{-\frac{\rho_l}{N}} + \frac{1-e^{-\frac{\rho_l}{N}}}{N}\right)\left(\frac{1-e^{-\frac{\rho_l}{N}}}{N}\right) & if\ one\ haplotype\ changes, but\ not\ the\ other \\ \left(\frac{1-e^{-\frac{\rho_l}{N}}}{N}\right)^2 & if\ both\ haplotypes\ change \end{cases} \tag{S3}$$

with $\rho_l = 4N_e r_l$, r_l being the per generation genetic distance between the sites l and $l+1$. The exponential dependence arises from the assumption of a Poisson process of recombination at any position in the genome, while the division by N in the exponent ensures that the probability of a recombination event drops exponentially with growth in the reference haplotype database. The latter idea ensures that the number of truly novel haplotypes reaches a plateau, i.e. there is a stable set of possible haplotypes in a population. In terms of the recombination rate c_l and the physical distance between adjacent loci $d_{l\to l+1}$, $r_l = c_l \times d_{l\to l+1}$. N is the number of reference haplotypes, and N_e is the effective population size, taken to be 11,418 (Li and Stephens 2003; Marchini et al. 2007; The International HapMap Consortium 2003).

In our methods, the linear model $\rho_l = 4N_e c_l d_{l\to l+1}$ is included as the default. However, we allow for the inclusion of a user-defined model of recombination in its place. For example, if

there is a known recombination hotspot between two adjacent query sites, it would alter the probability of transitioning between reference haplotypes in the search space, and thus impact the best-fit haplotypes calculated by the method. The user can explicitly include a vector of recombination values to be used for the $L-1$ intervals between query sites.

Another essential aspect of Equation S3 is the implicit assumption of uniform transition probabilities. That is, in its current form, Equation S3 does not discriminate between transitions from one haplotype to any other. If, on the other hand, a model is to be constructed where different subgroups have distinct recombination rates at particular locations and/or are assumed to impacted by assortative mating then transition probabilities would be conditional based on membership in these subgroups. In this iteration of our model, we do not provide a framework of this nature, but such an update would simply require the inclusion of appropriate bias terms conditional on the memberships of the initial and final haplotypes. However, we wish to emphasize that maintaining uniform transition probabilities helps prevent biased interpretations of ethnic group membership and isolation, and allows for the broad intermixing of haplotypes known to have occurred throughout human history (Narasimhan et al. 2019).

The other term in Equation S1, $P\left(G_q \middle| Z_j^{(1)}, Z_k^{(2)}\right)$, quantifies the probability of observing the query genotypes given a particular set of underlying haplotypes. This probability helps constrain the haplotypes that are possible given the observed genotypes, allowing for the case where mutations or genotyping errors occur (as considered in IMPUTE(Howie et al. 2009)).

$$P\left(G_q \middle| Z_j^{(1)}, Z_k^{(2)}\right) = \prod_{l=1}^{L} P\left(G_{q,l} \middle| Z_{j(l),l}^{(1)}, Z_{k(l),l}^{(2)}\right) = \prod_{l=1}^{L} P\left(Z_{j(l),l}^{(1)} + Z_{k(l),l}^{(2)} \rightarrow G_{q,l}\right) \qquad \text{(S4)}$$

with $P\left(Z_{j(l),l}^{(1)} + Z_{k(l),l}^{(2)} \rightarrow G_{q,l}\right)$ determined by the number of sites that require mutation to match the observed genotypes. We follow the suggestion of the authors of IMPUTE to consider a background rate of base pair mutation θ that translates into a mutation rate per haplotype of $\lambda = \frac{\theta}{2(N+\theta)}$ under the assumption of a neutral coalescent tree for N haplotypes(Li and Stephens 2003; Marchini et al. 2007). However, it is possible for the user to explicitly augment the background rate θ with contributions from genotyping error, or to ignore the mutation rate altogether and set $\theta = \frac{2N\lambda}{1-2\lambda}$ such that λ is equal to the known genotyping error. Thus, our code allows for either θ (the ***thetamutationrate*** parameter) or λ (the ***lambdamutationrate*** parameter) to be set. The values of $P\left(Z_{j(l),l}^{(1)} + Z_{k(l),l}^{(2)} \rightarrow G_{q,l}\right)$ dependent on λ are shown in **Supplemental Table S1**.

To summarize, the aim is to figure out the contribution to the total probability of each of the haplotype combinations, by estimating $P\left(G_q \middle| Z_j^{(1)}, Z_k^{(2)}\right).P\left(Z_j^{(1)}, Z_k^{(2)} \middle| H\right)$ for all haplotype trajectories, and to maximize this probability.

Hidden Markov Model optimization

The problem of identifying the best-fit combination of haplotypes is well-suited to the framework of Hidden Markov models (HMMs) given the traditional treatment of the genome as a linear sequence of base pairs. In this understanding, meiotic recombination between loci does not occur between distant locations of a chromosome (as may occur, hypothetically, due to

consistent 3D folding of the chromosomes within the nucleus), but has a certain probability of occurring at every intermediate site between any pair of loci. Usually, the greater the distance between the loci, the higher is the probability that recombination will have occurred in an ancestor of the query genome, though the probability is not necessarily uniform across every site. It then becomes easy to associate HMM emission probabilities at genomic sites with mutation rates and HMM transition probabilities between latent haplotypes with recombination rates. Furthermore, in the above expressions first-order Markovian behavior is assumed, and the observed output genotype is seen to depend only on the underlying haplotypes at that site alone (so-called output independence). This constrains the type of HMMs considered here, but leaves open interesting future applications where such assumptions are relaxed.

Accordingly, the problem of identifying the best trajectory through haplotype space can be carried out using the Viterbi algorithm (Viterbi 1967). This method solves the problem of maximizing the probability of the trajectories through the latent space in time $O((N \times N)^2 L)$, where N is the number of possible haploid states, i.e. the number of reference haplotypes, $N \times N$ is the corresponding number of diploid states, and L is the number of observed loci:

$$Most\ likely\ trajectory = \underset{Z_j^{(1)}, Z_k^{(2)} \in \left\{Z_{j(l),l}^{(1,2)}\right\}_{l=1;j=1}^{l=L;j=N}}{\operatorname{argmax}} P\left(G_q \middle| Z_j^{(1)}, Z_k^{(2)}\right) . P\left(Z_j^{(1)}, Z_k^{(2)} \middle| H\right)$$

$$= \underset{Z_j^{(1)}, Z_k^{(2)} \in \left\{Z_{j(l),l}^{(1,2)}\right\}_{l=1;j=1}^{l=L;j=N}}{\operatorname{argmax}} P\left(G_q, Z_j^{(1)}, Z_k^{(2)} \middle| H\right) \quad \text{(S5)}$$

where expressions for the two probabilities are given in Equations S2, S3 and S4.

The Viterbi algorithm achieves a more efficient solution to the optimization problem in Equation S5 than the naïve search by recognizing that the overall optimization problem can be separated into separate optimization steps at each query site with the convenient iterative scheme:

$$P\left(\left\{G_{q,\delta}\right\}_{\delta=1}^{l}, \left\{Z_{j(\delta),\delta}^{(1)}, Z_{k(\delta),\delta}^{(2)}\right\}_{\delta=1}^{l-1}, Z_{j(l),l}^{(1)}, Z_{k(l),l}^{(2)} \middle| H\right) =$$

$$P\left(G_q \middle| Z_{j(l),l}^{(1)}, Z_{k(l),l}^{(2)}, H\right) . \max_{j(l-1),k(l-1)} \begin{bmatrix} P\left(\left\{G_{q,\delta}\right\}_{\delta=1}^{l}, \left\{Z_{j(\delta),\delta}^{(1)}, Z_{k(\delta),\delta}^{(2)}\right\}_{\delta=1}^{l-2}, Z_{j(l),l}^{(1)}, Z_{k(l),l}^{(2)} \middle| H\right) \times \\ P\left(Z_{j(l-1),l-1}^{(1)} \to Z_{j(l),l}^{(1)}, Z_{k(l-1),l-1}^{(2)} \to Z_{k(l),l}^{(2)} \middle| H\right) \end{bmatrix}$$

$$\Rightarrow v_l\big(j(l), k(l)\big) = EmissionProb\left(G_{q,l} \middle| Z_{j(l),l}^{(1)}, Z_{k(l),l}^{(2)}, H\right) \times$$
$$\max_{j(l-1),k(l-1)} \left[v_{l-1}\big(j(l-1), k(l-1)\big) . TransitionProb\left(Z_{j(l-1),l-1}^{(1)} \to Z_{j(l),l}^{(1)}, Z_{k(l-1),l-1}^{(2)} \to Z_{k(l),l}^{(2)} \middle| H\right)\right]$$

with $v_l\big(j(l), k(l)\big) = P\left(\left\{G_{q,\delta}\right\}_{\delta=1}^{l}, \left\{Z_{j(\delta),\delta}^{(1)}, Z_{k(\delta),\delta}^{(2)}\right\}_{\delta=1}^{l-1}, Z_{j(l),l}^{(1)}, Z_{k(l),l}^{(2)} \middle| H\right)$

(S6)

where the algorithm initializes a value of $v_1\big(j(1), k(1)\big)$ and then proceeds to iteratively update the probability. The second line of Equation S6 simply provides a clearer conceptual understanding of the first line.

$P\left(\left\{G_{i,\delta}\right\}_{\delta=1}^{l}, \left\{Z_{j(\delta),\delta}^{(1)}, Z_{k(\delta),\delta}^{(2)}\right\}_{\delta=1}^{l-1}, Z_{j(l),l}^{(1)}, Z_{k(l),l}^{(2)} \middle| H\right)$ is the joint probability of the observed genotypes at all sites up to and including site l, and of the reference haplotypes at all sites up to site $l-1$, with the reference haplotypes at site l being fixed at $Z_{j(l),l}^{(1)}, Z_{k(l),l}^{(2)}$, and is easily related to the argument of the *argmax* function on the right-hand side of Equation S5.

Matrix methods used in the modified Viterbi algorithm. The probability vectors were encoded as Python *numpy* arrays. Assuming an unbiased transition matrix (Equation S3, no assumed subpopulation membership), each *argmax* calculation in Equation 2 was calculated over an array whose elements were updated as follows:

a. Let $\log v_l(j,k)$ be the log-probability vector (Equation S6).
b. $\log v_l(j,k)$ is a matrix indexed by every pair of reference haplotypes. This matrix was flattened in 1D, keeping only the lower triangle of the matrix.
c. At each observed genotype locus, initialize $\log v_l(j,k) = \log E_l^{G_l}(j,k)$, the vector of precalculated log-emission-probabilities for each pair of reference haplotypes and the observed genotype.
d. For $l=1$, set $\log v_1(j,k) = \log \frac{E_1^{G_1}(j,k)}{N^2}$, with the assumption of equal likelihood of all reference haplotypes at the first observed locus.
e. Define $T_{on} = \log\left(e^{-\frac{\rho_l}{N}} + \frac{1-e^{-\frac{\rho_l}{N}}}{N}\right)$ and $T_{off} = \log\left(\frac{1-e^{-\frac{\rho_l}{N}}}{N}\right)$ (the log probabilities of retaining a haplotype and crossing over to another one, respectively; see Equation S3).
f. Define the matrix $\Delta(j,k) = \log v_{l-1}(j,k) + 2T_{off}$
g. For every pair of reference haplotypes (j,k), update all elements in the same row and column: $\Delta(j,.) \mathrel{+}= T_{on} - T_{off}$ and $\Delta(.,k) \mathrel{+}= T_{on} - T_{off}$.
h. Find the maximum log-probability $M(j,k) = \max_{i,j} \Delta(i,j)$ and the corresponding arguments $BT(j,k) = \operatorname{argmax}_{\alpha,\beta} \Delta(\alpha,\beta)$, where the last term is the backtrace vector.
i. Repeat steps (f)-(h) for all haplotype pairs. The matrix $\Delta(j,k)$ is reinitialized every time in step (f).
j. Importantly, we further modified the previous step to include all pairs of haplotypes that were within a certain range of the absolute maximum (by default, the cutoff was $|M(j,k)| - 0.01 * |M(j,k)|$). We did this as, given the assumed data sparsity, it was likely that multiple haplotypes would match exactly or nearly so. Additionally, this looser definition of maximization also compensates any rounding-off errors that may cause two similar paths to diverge in log-probability. Changing this parameter could also allow the user to discover sub-optimal paths.
k. Update $\log v_l(j,k) \mathrel{+}= M(j,k)$.
l. Repeat steps (c)-(k) for every observed genotyped site.
m. When $l=L$, the process is terminated by finding $M = \max_{\alpha,\beta} \log v_L(\alpha,\beta)$ and $BT = All\ (j,k)\ such\ that\ |\log v_L(j,k)| \geq |M| - 0.01 * |M|$.

1) Using this terminal set of BT, the corresponding backtrace values for each selected reference haplotype pair are traced all the way back to the first observed locus. This results in a set of trajectories that may fork and merge.
2) Instead of explicitly storing the backtrace vector in RAM, we use the Python package *gzip* to write the backtrace vector for each observed site directly to a gzipped file. We similarly read through the gzipped file during the final stage of reading out the best-fit trajectories.

Truncation scheme. In the *PLIGHT_Truncated* module, at every observed site we truncate the possible haplotype states by choosing the top T sets of (α, β) pairs. This scheme was inspired by similar techniques employed in the Eagle2 imputation program (Loh et al. 2016). The main premise of this approximation is after a certain point, only a fraction of a the total number of trajectories will meaningfully contribute to the best-fit states, and allow the retention of only a fraction of the total number of states in memory. However, in addition to tracking the probabilities and backtrace vectors of these states, we now also need to retain positional indices associated with the particular pairs for future look-up (i.e. we need to know the identity of these pairs).

The module allows the user to set the truncation factor as the fraction of the total number of reference haplotype pairs retained in the calculation:

Truncation factor $f = T/\left(\frac{N(N+1)}{2}\right) = T/T_{tot}\,;\ T_{tot} = \frac{N(N+1)}{2}$ (S7)

However, we recognize that immediate truncation at the first location would not allow the probabilities of the most likely trajectories to build up sufficiently. Therefore, we phase-in the truncation by using a linear decrease in the value of T until the halfway point:

$$T_l = \begin{cases} floor(slope \times (l-1) + T_{tot}), for\ l \leq floor\left(\frac{L}{2}\right) \\ floor(f \times T_{tot}), for\ l > floor\left(\frac{L}{2}\right) \end{cases} \quad \text{(S8)}$$

where $slope = T_{tot} \times (f-1)/\left(floor\left(\frac{L}{2}\right) - 1\right)$ and $floor(x)$ is the rounding down function. Furthermore, setting a cutoff on the number of states could possibly arbitrarily remove states that have the same probability as the included ones. Accordingly, we soften the truncation by including all additional states that have the exact same probability as the final T_l state. Note that this may grow the size of the matrices significantly, if the number of equiprobability or degenerate states is large. This may occur for very sparse data.

The advantage of the truncation scheme is the reduction of the size of the matrices. To maintain this advantage, we had to explicitly calculate the probabilities at the first observed site, where the truncation had not been applied yet. This prevents (in the current version of the code) the inclusion of missing genotypes. Additionally, the parallelization procedure at the first observed site required the chunking up of the haplotype pairs into subgroups the size of $floor(f \times T_{tot})$, and running each of these subgroups through parallel matrix calculations. This prevented the necessity of having the entire set of haplotype pairs be manipulated in matrices simultaneously, which would have defeated the purpose of the truncation process.

Imputation of blood tissue sample genotypes

The blood tissue sample genotypes were phased using the TOPmed Imputation Server(Taliun et al. 2021; Das et al. 2016; Fuchsberger et al. 2015). Specifically, we used the TOPmed reference panel version r2, with an array build of GRCh37/hg19 for the unphased VCF file; the "rsq Filter" was set to "off"; the phasing used "Eagle v2.4"; no "Population" was selected; and we ran the server in "Quality Control & Phasing Only" mode. Since, the output of the TOPmed phasing was aligned to hg38, we used the *LiftoverVcf* from Picard tools (Broad Institute) to lift over the variants to the hg19 reference, so as to match up with the existing 1000 Genomes-based vcf files.

Mosaic genome correspondence and polygenic risk scores

Correspondence score. Using mosaic genome reconstructions, we assessed the accuracy to which we could impute SNPs across the genomic region covered by the observed SNP loci. The accuracy metrics included a straightforward calculation of the fraction of SNPs exactly matched in genotype dosage, as well as a measure of the degree to which the inferred trajectory matched the query genome, with the contribution from each SNP weighted by a function of the genotype frequency:

$$Correspondence\ score\ C \equiv \frac{1}{N_{SNP}}\sum_{s=1}^{N_{SNP}}\left(1-p_s(G_s^{\mathcal{T}})\right).\frac{\left(2-\left|G_s^{\mathcal{T}}-G_s^{Q}\right|\right)}{2} \qquad \text{(S9)}$$

C measures the total correspondence between the set of query individual genotypes, $\{G_s^Q\}_{s=1}^{N_{SNP}}$, and the set of genotypes for trajectory $\mathcal{T}$, $\{G_s^{\mathcal{T}}\}_{s=1}^{N_{SNP}}$, where N_{SNP} is the total number of overlapping SNPs defined in the VCFs of the reference database and the query individual between the first and last observed SNPs. Next, $\left|G_s^{\mathcal{T}}-G_s^{Q}\right|$ quantifies the deviation of the genotype dosage of $\mathcal{T}$ from that of the query Q at SNP position s, and is subtracted from 2 and divided by 2 to set a score scale where 0 corresponds to maximal deviation of $\mathcal{T}$ from Q and 1 corresponds to a perfect match between the two. Finally, $p_s(G_s^{\mathcal{T}})$ is the genotype frequency (as opposed to the allele frequency) of the SNP dosage $G_s^{\mathcal{T}}$, which is the probability that trajectory $\mathcal{T}$ could have a given dosage at random based on population occurrence frequencies. The heuristic $\left(1-p_s(G_s^{\mathcal{T}})\right)$ is therefore a measure of the non-randomness of the trajectory SNP dosage. $C = 0$ when no SNPs match between $\mathcal{T}$ and Q and/or the SNPs occurred in the reference population at 100% frequency, while $C \approx 1$ when $\mathcal{T}$ and Q agree at every SNP position and the SNPs are extremely rare (and so the matching of the two is very likely to be a non-random occurrence).

We compared the fraction of correct SNPs and the correspondence scores for our trajectories to the equivalent scores calculated on a set of 99 randomly selected genomes in the same genomic regions from the 1000 Genomes cohort.

Polygenic risk score calculation. We perform approximate calculations of the linear polygenic risk scores (PRSs) based on all SNP associations in the GWAS catalog version 1.0.2 (Buniello et al. 2019). We first identify all individuals in the trajectories of each HMM run. We then constructed the (previously described) diploid mosaic genome of the query individual based on each trajectory. The resulting genotypes are used to calculate PRSs for each phenotype y and each individual n as:

$$PRS(y,n) \equiv \sum_{i=1}^{R(y)} \beta_i x_i^n, \quad \text{(S10)}$$

where $\beta_i = Signed\ effect\ size\ of\ the\ risk\ allele\ at\ SNP\ i$,
$x_i^n \in \{0,1,2\} = Genotype\ of\ individual\ n\ at\ SNP\ i$,
and $R(y) = Total\ number\ of\ risk\ alleles\ for\ phenotype\ y$.

The PRS is very approximate in the sense that no SNP filtering was conducted beyond those presented in the GWAS catalog, either by p-value or by LD with other associated SNPs. However, the aim here is merely to determine whether there are aggregate properties across the genome that can be inferred using our approach. We calculated the Pearson's correlation between the PRSs of the true samples and the best-fit mosaic genomes within the regions and chromosomes sampled. All traits for which the PRS of the true sample was non-zero ('non-zero traits') were included. To assess whether the PRS correlations between the true individual and inferred mosaic genomes were statistically significant, we sampled a background set of ~100 individuals from the 1000 Genomes dataset that did not occur in any of the test sets we ran, and calculated the PRSs for the non-zero traits.

We employed several statistical metrics to assess the correspondence in PRSs between the query genomes and the best-fit mosaic genomes, relative to the background scores: (1) the cosine similarity between the query and mean values of the best-fit scores, compared to the mean value of the background scores; (2) the same metric as in (1) but only for traits with more than one GWAS SNP in the regions sampled (thus removing one-SNP traits); (3) the same metric as in (1) but only for traits where the query PRS had an absolute *Z*-score > 2 (i.e. traits for which the query itself is an outlier relative to the background); and (4) the cosine similarity between the query genome and each of the best-fit mosaic genomes, followed by the mean of all the cosine similarity values (same for the background).

Supplemental Results

Simulation of the degree of correlation of randomly selected SNPs.

As described in the Supplemental Methods, the goal of this analysis was to explore the degree of SNP correlation that occurs even when randomly sampling SNPs across the entire genome (one of the possible cases where PLIGHT would be useful). We tested out the longest chromosome, Chromosome 1, and the shortest autosomal chromosome, Chromosome 22, with 30 randomly selected SNPs, as well as Chromosome 1 with 90 randomly selected SNPs. Shown below are the number of SNP pairs found with a linkage disequilibrium (LD) R^2 > 0.5, where we tested the LD score against 5 superpopulations "AFR","AMR","EAS","EUR","SAS" in the 1000 Genomes cohort (The 1000 Genomes Project Consortium 2015). It is possible for pairs to be counted in the LD analysis for a single superpopulation, or across multiple superpopulations (eg., a single SNP pair could be under LD in both the AFR and SAS superpopulations, and this would count as two pairs in total). The results are:

Chromosome 1 (the longest chromosome), N = 30 : 41 out of the 1,000 simulations produced a pair of correlated SNPs, with 3 cases of 2 pairs being found.

Chromosome 22 (the shortest autosomal chromosome), N = 30 : 31 out of the 1,000 simulations produced a pair of correlated SNPs, with 1 case of 2 pairs, 3 pairs, 4 pairs and 5 pairs each being found.

Chromosome 1, N = 90 : 236 out of the 1,000 simulations produced a pair of correlated SNPs, with 45 cases of 2 pairs, 14 cases of 3 pairs, 6 cases of 4 pairs and 1 case of 5 pairs being found.

These results indicate that, while a very small number of SNPs randomly selected do not exhibit much correlation structure, the degree of correlation goes up with an increase in the number of SNPs. The degree would be higher if we allow for more moderate LD (i.e. if we lower the R^2 cutoff to a value below 0.5). In general, given that an attack on privacy would not have to follow the random selection process (as discussed in the paper), we foresaw a need for a more general model of SNP matching.

Supplement to Section "Identification of individuals known to be within a database".

We examine the reasons for the non-monotonic behavior of the $N_{SNP}^{Correct}$ values in Table 1. We run an independent simulation to the one in Table 1, using the same parameters: chose 10 individuals, one at a time, from the 1000 Genomes cohort; for each individual, ran 40 separate SNP selection runs, corresponding to $N_{SNP} \in [1, \cdots, 40]$; select SNPs according to a Bernoulli process with probability 0.003 until we reach the chosen N_{SNP} value; mutate each SNP's genotype as a function of the mutation rate λ (according to **Supplemental Table S1**), and include the SNP if the mutated genotype is either heterozygous or homozygous in the alternate allele. This time we run simulations up to higher mutation rates: 0.0, 0.05, 0.1, 0.2, 0.3, 0.4, 0.5, 0.6. We then run *PLIGHT_InRef* on these query sets. For the runs where *PLIGHT_InRef* successfully found the correct query individual, we count the total number of individuals (including the correct one) found in the best-fit trajectories across all 10 query individuals and all SNP numbers as a function

of the mutation rate. The results are shown in **Supplemental Fig. S2**. Given that the metric of interest in Table 1 is $N_{SNP}^{Correct}$, the number of SNPs needed for both correct and unique identification, we can see that the distributions up to $\lambda = 0.2$ are mostly concentrated close to 1. This implies that there are a large number of runs for low mutation rates that have correctly and uniquely identified the query individual. On the other hand, as λ increases to 0.3, the tail of the distribution is at 1, while for $\lambda > 0.3$, there are no instances of correct and unique identification. The numbers of runs with correct and unique identification at each mutation rate are: $\lambda = 0.0$, 334 runs; $\lambda = 0.05$, 285 runs; $\lambda = 0.1$, 222 runs; $\lambda = 0.2$, 62 runs; $\lambda = 0.3$, 8 runs. When $N_{SNP}^{Correct}$ as the average *minimum* number of SNPs required for a correct and unique identification, we find

$N_{SNP}^{Correct} = 6.8 \pm 1.5$ across 10 individuals, for $\lambda = 0.0$
$N_{SNP}^{Correct} = 8.3 \pm 2.0$ across 10 individuals, for $\lambda = 0.05$
$N_{SNP}^{Correct} = 9.9 \pm 2.6$ across 10 individuals, for $\lambda = 0.1$
$N_{SNP}^{Correct} = 19.7 \pm 6.1$ across 10 individuals, for $\lambda = 0.2$
$N_{SNP}^{Correct} = 27.0 \pm 13.0$ across 5 individuals, for $\lambda = 0.3$

We see that the number is less stable for $\lambda = 0.3$ (where only 5 individuals had the requisite identifications), and that the monotonic trend is recovered.

Supplement to Section "Identification of individuals from contaminated samples". We examine the reasons for the non-monotonic behavior of the $N_{SNP}^{Correct}$ values as a function of the replacement rate in the contamination study in Table 2. We run replicate simulations using the same methods as described in the main manuscript, except for the fact that we set the error rate to be 0.0. The results, shown in **Supplemental Fig. S3**, indicate that when no possibility of error is included in the PLIGHT model, there is a monotonic increase (as a function of the replacement rate) in the number of possible individuals who are found along with the correct individuals. In other words, there is a steady increase in the difficulty of uniquely picking out the query individual. As discussed in the main manuscript, a larger input error rate in the inference allows greater leeway in the overlap between the observed SNPs and the reference database individuals. This is the reason for the observed increase in the number of unique and correct identifications at higher replacement rates. Thus, comparing the results in the main manuscript and those in the replicate analysis here, we suggest that the inference process may improve by increasing the allowed error in the model.

Supplement to Section "Truncated algorithm". We ran the same SNP set as in Section "***Exact search within a reference database of 400 haplotypes***" through the truncated algorithm to assess the degree of compressibility of the trajectories, with a recombination rate of 0.5 cM/Mb and with a search through 200 reference individuals. In interpreting the results, it is worth bearing in mind the fact that while the algorithm was set up to slowly phase-in the restriction of the number of considered haplotype pairs to the top few, it yet retained a degree of elasticity to prevent an abrupt cut-off: all haplotype pairs with the same probability as the last state in the imposed cut-off were also included (**Supplemental Fig. S6**). We ran the calculation for two different asymptotic truncation levels, with the truncation factor f in Equations S7 and S8 = 0.005

and 0.02 (**Figs. S6A** and **S6B**). For reference, the fractions of the matrix sizes at each SNP in the calculations are shown for f = 0.005 (**Fig. S6A**) and f = 0.02 (**Fig. S6B**). The resultant trajectories for Chromosome 1 were identical to the exact algorithm, implying that reducing the number of considered trajectories did not impact the search process. For Chromosome 21, the lower truncation level of 0.005 resulted in fewer trajectories being included, while the higher truncation of 0.02 reproduced the same trajectories as the exact algorithm. Chromosome 2 appeared to be less resilient to truncation and produced different results from the exact algorithm (the trajectories for f = 0.005 are shown in **Fig. S6C**; compare to **Fig. 3B**). Note that truncation at later SNPs favors the prioritization of HG00360 in the earlier parts of the chromosome, whereas the exact algorithm with the same recombination rate removes this individual from the final results. The degree to which different trajectories are resilient to truncation is a measure of the degree to which the best trajectories separate out from the others. In informatic terms, if the best trajectory probabilities (analogous to the energies of physical states) have low entropy (analogous to deep, sharp valleys in the energy landscape) trajectory truncation will not impact the search; if the entropy is high (broad, shallow valleys in the energy landscape) truncation will impact the results.

Supplement to “Predicting genotypes at GWAS loci and polygenic risk score (PRS) analysis”

We construct a query set of 90 SNPs, each within ± 2 kb of known GWAS SNPs for the phenotype “Height”. This increases the likelihood of LD with GWAS variants. Within these windows, 25 GWAS variants are found for Chromosome 3 and 26 for Chromosome 6. We ran *PLIGHT_Iterative* with $n_{iter} = 20$ and $S_{sg} = 300$ on the query sets, and study the resulting 5 and 12 trajectories for Chromosomes 3 and 6, respectively. First, we looked at the 22 and 23 GWAS SNPs in Chromosomes 3 and 6, respectively, not directly in the query sets and checked how well the inferred trajectories matched the unseen query genotypes. For Chromosome 3, the 5 trajectories produced $\{10, 10, 10, 13, 13\}$ matches out of 22 SNPs. We also checked the same SNP sites for 100 randomly sampled individuals, and ran a Welch’s two-sided, two-sample *t*-test between the two distributions, obtaining a p-value of 0.004. However, a few of the background samples matched the GWAS SNPs to a higher degree (16 SNPs out of 22). We therefore looked at the consensus across the trajectories and found that the 5 trajectories matched the query genotypes perfectly at 9 GWAS SNP sites, and were off by a dosage of 1 at the remaining sites. The background individuals had mixed results across all SNP sites. The trajectories for Chromosome 6 yielded match rates of at most 12 out of 23 and a two-sided, one-sample *t*-test with respect to the background distribution for the same 100 individuals yielded a p-value of 0.56. Many of the background individuals did better than 12 out of 22 matches. Thus, the imputation of GWAS SNPs did not indicate that the inferred trajectories do better at imputing the GWAS SNPs.

We also assessed whether the inferred PRSs are a better match than a background set of PRSs from 100 randomly sampled individuals. We consider the statistical significance of the deviation from the true sample’s PRS (a Welch’s two-sided, two-sample *t*-test). The resulting p-values for the absolute PRS deviations: Chromosome 3 = 0.25, and Chromosome 6 = 0.15. In both cases, the trajectory-based PRSs were not significantly closer to the true sample’s PRS than the mean of the background PRSs (distributions in **Supplemental Fig. S9A-B**).

Supplement to “Sanitization of SNPs based on Inferred Trajectories”

We address specific examples of scenarios where published data may be subject to the type of sanitization considered in our work.

1. There are cases where DNA samples from environmental objects may be published, such as for DNA from historical objects (eg. ongoing work from co-authors of this paper, https://www.nyhistory.org/blogs/extracting-stories-from-19th-century-dna), which may also be contaminated with DNA from living individuals. Any such published data can be sanitized using the method described.
2. Second, another possible situation where publication is an issue is the production of functional genomics data where a limited number of SNPs may be extracted, such as microarray data from targeted sites.
3. Finally, for nanopore reads, we envisioned using PLIGHT for the case where considerable contamination from multiple individuals may obscure the provenance of multiple reads. On the other hand, a single read will undoubtedly be derived from a single individual, and so it would be worthwhile running the sanitization process on even a single read’s SNPs to prevent significant identifying information regarding that individual.

References

Broad Institute. Picard Tools. http://broadinstitute.github.io/picard/.

Buniello A, Macarthur JAL, Cerezo M, Harris LW, Hayhurst J, Malangone C, McMahon A, Morales J, Mountjoy E, Sollis E, et al. 2019. The NHGRI-EBI GWAS Catalog of published genome-wide association studies, targeted arrays and summary statistics 2019. *Nucleic Acids Res* **47**: D1005–D1012.

Das S, Forer L, Schönherr S, Sidore C, Locke AE, Kwong A, Vrieze SI, Chew EY, Levy S, McGue M, et al. 2016. Next-generation genotype imputation service and methods. *Nat Genet* **48**: 1284–1287. https://doi.org/10.1038/ng.3656.

Fuchsberger C, Abecasis GR, Hinds DA. 2015. minimac2: faster genotype imputation. *Bioinformatics* **31**: 782–784. https://doi.org/10.1093/bioinformatics/btu704.

Howie BN, Donnelly P, Marchini J. 2009. A flexible and accurate genotype imputation method for the next generation of genome-wide association studies. *PLoS Genet* **5**.

Li N, Stephens M. 2003. Modeling Linkage Disequilibrium and Identifying Recombination Hotspots Using Single-Nucleotide Polymorphism Data. *Genetics* **165**: 2213–2233.

Loh PR, Danecek P, Palamara PF, Fuchsberger C, Reshef YA, Finucane HK, Schoenherr S, Forer L, McCarthy S, Abecasis GR, et al. 2016. Reference-based phasing using the Haplotype Reference Consortium panel. *Nat Genet* **48**: 1443–1448.

Marchini J, Howie B, Myers S, McVean G, Donnelly P. 2007. A new multipoint method for genome-wide association studies by imputation of genotypes. *Nat Genet* **39**: 906–913.

Myers TA, Chanock SJ, Machiela MJ. 2020. LDlinkR: An R Package for Rapidly Calculating Linkage Disequilibrium Statistics in Diverse Populations. *Front Genet* **11**. https://www.frontiersin.org/articles/10.3389/fgene.2020.00157.

Narasimhan VM, Patterson N, Moorjani P, Rohland N, Bernardos R, Mallick S, Lazaridis I, Nakatsuka N, Olalde I, Lipson M, et al. 2019. The formation of human populations in South and Central Asia. *Science* **365**.

Taliun D, Harris DN, Kessler MD, Carlson J, Szpiech ZA, Torres R, Taliun SAG, Corvelo A, Gogarten SM, Kang HM, et al. 2021. Sequencing of 53,831 diverse genomes from the NHLBI TOPMed Program. *Nature* **590**: 290–299. https://doi.org/10.1038/s41586-021-03205-y.

The 1000 Genomes Project Consortium. 2015. A global reference for human genetic variation. *Nature* **526**: 68–74.

The International HapMap Consortium. 2003. The International HapMap Consortium. The International HapMap Project. *Nature* **426**: 789–796.

Viterbi A. 1967. Error bounds for convolutional codes and an asymptotically optimum decoding algorithm. *IEEE Trans Inf Theory* **13**: 260–269.
